# Biogeochemical feedbacks associated with the response of micronutrient recycling by zooplankton to climate change

**DOI:** 10.1111/gcb.15789

**Published:** 2021-07-29

**Authors:** Camille Richon, Alessandro Tagliabue

**Affiliations:** ^1^ School of Environmental Sciences University of Liverpool Liverpool UK; ^2^ Present address: Laboratoire d'Océanographie Physique et Spatiale UMR 197 CNRS/IFREMER/IRD/UBO Institut Universitaire Européen de la Mer Plouzané 29280 France

**Keywords:** biogeochemistry, climate change, micronutrients, recycling, zooplankton

## Abstract

Recycling by zooplankton has emerged as an important process driving the cycling of essential micronutrients in the upper ocean. Resupply of nutrients by upper ocean recycling is itself controlled by multiple biotic and abiotic factors. Although the response of these drivers to climate change will shape future recycling rates and their stoichiometry, their magnitude and variability are unaddressed by climate change projections, which means potentially important feedbacks on surface biogeochemistry are neglected. Here, we assess the impacts of climate change under the high emissions RCP8.5 scenario on the recycling of the essential micronutrients Fe, Zn, Cu, Co and Mn and quantify the regional control by zooplankton food quality, prey quantity, sea surface temperature and zooplankton biomass. A statistical assessment of our model results reveals that the variability in recycling fluxes across all micronutrients is mainly driven by the variability of zooplankton and prey biomass. In contrast, the variability in micronutrient recycling stoichiometry and its response to climate change are more complex and is regulated by zooplankton food quality and prey quantity. Regionally, the relative influence of each driver on recycling changes in our model by the end of the 21st century. Temperature becomes an important driving factor in the polar regions while the expansion of oligotrophic regions leads to the importance of food quality increase for low and mid‐latitudes. These responses lead to novel feedbacks that can amplify the response of surface ocean biogeochemistry and alter nutrient deficiency regimes.

## Graphical abstract

Micronutrient recycling by zooplankton is an important biogeochemical process that is influenced by different biotic and abiotic drivers. Here, we assess climate change impacts on micronutrient recycling fluxes and stoichiometry and their different drivers. A statistical assessment of our model results reveals that the variability in recycling fluxes is mainly driven by the variability of zooplankton and prey biomass whereas the variability in recycling stoichiometry and its response to climate change is more complex and regulated by zooplankton food quality and prey quantity. The feedbacks from changing recycling on surface biogeochemistry depend on the complex interaction between recycling drivers.

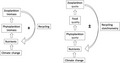

## 1 INTRODUCTION

Trace metals are essential micronutrients for marine phytoplankton and their scarcity in the environment can contribute to regulating primary productivity and ecosystem functioning in the surface ocean (Moore et al., 2013; Tagliabue et al., 2020), as well as being toxic at high concentrations (Brand et al., 1986; Debelius et al., 2011). A range of micronutrients have various essential roles in phytoplankton and zooplankton physiology and therefore affect the ocean cycles of major elements, such as carbon (C), nitrogen (N), silica (Si) and phosphorus (P; Morel et al., 2014). The relative amount of micronutrients in phytoplankton primary producers is known as the stoichiometry and is much more variable (3‐ to 20‐fold) for micronutrients than for major elements, such as N and P (Twining & Baines, 2013). Zooplankton grazers rely on the micronutrient content of their ingested prey to satisfy their nutritional requirements and after assimilation, any excess nutrients are directly excreted in the dissolved phase at the surface or egested as faecal pellets, which sink to depth (see Richardson, 2008; Steinberg & Landry, 2017).

Micronutrient concentrations in the upper ocean are generally very low (in the micro to picomolar range) and recycling by zooplankton has emerged as an important component of upper ocean micronutrient cycling in prior work. Recycling has been shown to play a crucial role for Fe (Boyd et al., 2015; Sarthou et al., 2008; Strzepek et al., 2005; Tagliabue et al., 2014) and under resource limitation, zooplankton recycling can sustain phytoplankton activity (see Laglera et al., 2017; Rafter et al., 2017). In this context, recycling of dissolved micronutrients by zooplankton increases their surface ocean residence time (Boyd et al., 2012; Hutchins & Bruland, 1994). The importance of micronutrient recycling by zooplankton stems from two main mechanisms. First, the mismatch between zooplankton and prey micronutrient stoichiometries can lead to high rates of recycling due to altered zooplankton assimilation efficiencies (Richon et al., 2020). Second, the reduced supply of micronutrients from external sources over much of the open ocean (Gaillardet et al., 2014; Mahowald et al., 2018) makes internal cycling a key driver of upper ocean inventories.

Nutrient recycling by zooplankton is influenced by many direct and indirect factors that are under both biotic and abiotic control. Temperature directly impacts zooplankton distributions, their growth and a range of physiological rates (Richardson, 2008), as well as the underlying ocean circulation. Zooplankton biomass is a major driver of micronutrient recycling rates, as a greater abundance of zooplankton will lead to greater recycling fluxes. The amount of ingested prey affects recycling through its effect on nutrient assimilation, with greater overall prey ingestion leading to a reduced zooplankton assimilation efficiency and higher recycling rates (Richon et al., 2020; Xu & Wang, 2001). Finally, the absolute and relative micronutrient availability in the surface ocean influences phytoplankton uptake, which drives the phytoplankton micronutrient stoichiometry, which, in turn, impacts recycling by controlling food quality (Richon et al., 2020). Food quality was defined by Richon et al. (2020) as the dimensionless balance between the zooplankton stoichiometry and the stoichiometry of their ingested prey. When food quality is close to 1, zooplankton are feeding on stoichiometrically similar prey. If food quality deviates from 1, then prey are either richer or depleted in nutrients, which leads to excess or insufficient ingestion by zooplankton. A food quality factor close to 1 favours low recycling due to a high assimilation efficiency, while as food quality moves further away from 1, recycling increases as the assimilation efficiency drops (Richon et al., 2020). Despite its impacts on surface biogeochemistry, recycling is challenging to quantify in situ (e.g. Boyd et al., 2012). This means that the impacts of this range of biotic and abiotic drivers on zooplankton recycling and the biogeochemical and ecological consequences are difficult to identify (Mayzaud & Pakhomov, 2014). In this context, global ocean models can be useful as they represent various abiotic and biotic drivers of zooplankton recycling and their role in driving modelled recycling fluxes and stoichiometry can be assessed.

The consequences of climate change on ocean surface circulation, nutrient and ecosystem structure and function have been studied extensively (e.g. Bindoff et al., 2019; Kwiatkowski, Aumont, & Bopp, 2018; Kwiatkowski, Aumont, Bopp, & Ciais, 2018; Kwiatkowski et al., 2020; Lotze et al., 2019). Increased stratification due to climate change affects vertical nutrient supply and alters the strength of phytoplankton nutrient limitation (e.g. Tagliabue et al., 2020). These changes will also affect micronutrient uptake and phytoplankton stoichiometry while zooplankton recycling rates will respond to the integrated effect of the full suite of drivers. Temperature, plankton biomass and food quality may vary in different directions across different micronutrients and in distinct regions due to their unique cycling and distinct biological requirements. This means that the impacts of climate change on recycling fluxes and stoichiometry are poorly quantified and absent from climate change assessments of ecosystem change (Bindoff et al., 2019). Due to the key role of recycling for surface ocean biogeochemistry, important feedbacks that may operate between climate‐driven changes in micronutrient recycling and resource availability for upper ocean ecosystems have been neglected.

In this article, we use a global coupled physical‐biogeochemical model under a high greenhouse gas emissions scenario (RCP8.5) to assess the climate change impacts on surface micronutrient biogeochemistry. We focus on iron (Fe), cobalt (Co), copper (Cu), manganese (Mn) and zinc (Zn), included in an earth system model for the first time. Our results show that recycling fluxes decrease for all micronutrients in the low latitudes and increase in the mid‐ to high latitudes by the end of the 21st century. On the other hand, recycling stoichiometry (Micronutrient:C ratio in recycled material) varies depending on the metal. Then, we explore the changes in recycling drivers (zooplankton food quality, prey quantity, sea temperature and microzooplankton biomass) and use statistical models to identify how different driving factors influence micronutrient recycling in different ocean regions in the beginning and by the end of the century. Finally, we discuss how the changes in the different drivers may impact recycling and how this may feedback on surface micronutrient biogeochemistry.

## 2 METHODS

### 2.1 The NEMO‐PISCES model and experimental approach

We use the global coupled physical‐biogeochemical model NEMO/PISCES (Aumont et al., 2015), which represents nitrate, ammonium, phosphate, silicic acid and dissolved iron cycling, the full carbon and oxygen systems, two phytoplankton groups (nanophytoplankton and diatoms) and two zooplankton size classes (microzooplankton and mesozooplankton) and has been extensively used to study regional and global ocean biogeochemistry (e.g. Aumont et al., 2017; Gorgues et al., 2019; Kwiatkowski, Aumont, Bopp, & Ciais, 2018; Richon et al., 2017; Tagliabue & Resing, 2016). Recent developments of the PISCES model have included micronutrient cycling such as Cu (Richon & Tagliabue, 2019), Zn, Co (Tagliabue et al., 2018) and Mn to build a new version of the model called PISCES‐BYONIC. The model is fully described and evaluated in supplement (Text S1). We present here the key information on the model.

In the standard version of PISCES‐BYONIC, the phytoplankton macronutrient stoichiometry (C:N:P) is fixed, but it is variable for micronutrients (Fe, Co, Cu, Mn and Zn), chlorophyll and silica. The maximum micronutrient:C molar quotas are 80E‐6 for Fe, 40 and 123 for Zn in nanophytoplankton and diatoms, respectively, 16E‐6 for Cu, 1.2E‐6 for Co and 8E‐6 for Mn, which broadly reflects available observational constraints (e.g. Twining & Baines, 2013; Twining et al., 2015). The zooplankton molar micronutrient to carbon stoichiometry is fixed to 10E‐6 for Fe, Zn and Cu, and to 0.16E‐6 and 1E‐6 for Co and Mn, respectively, following the more limited observational understanding (see Baines et al., 2016; Ratnarajah et al., 2014; Twining & Baines, 2013).

The impacts of climate change on micronutrient recycling and recycling stoichiometry were simulated using offline physical fields from the IPSL‐CM5A climate model, as in previous work (Kwiatkowski, Aumont, Bopp, & Ciais, 2018; Tagliabue et al., 2020). We performed two simulations: a preindustrial control (PICONTROL) from 1801 to 2100 with atmospheric CO_2_ concentrations fixed to the preindustrial value. Then, from 1851 to 2100, we performed a second simulation initialized from year 1851 of the PICONTROL, with CO_2_ concentrations varying according to the historical pathway until 2005 and switching to the high emissions RCP8.5 scenario (Riahi et al., 2011) from 2006 to 2100. Previous studies using NEMO/PISCES under the RCP8.5 scenario showed a global increase in stratification, increased SST and decrease in surface macronutrients leading to changes in plankton distribution and stoichiometry (Kwiatkowski, Aumont, & Bopp, 2018; Kwiatkowski, Aumont, Bopp, & Ciais, 2018).

For our simulations, we use constant external nutrient sources (hydrothermal vents, rivers and aerosols). Sedimentary sources of Co and Mn are O_2_ dependent (Tagliabue et al., 2018). To assess our results, we define two periods of time: PRESENT (model results averaged over 1991–2000) and FUTURE (model results averaged over 2091–2100). Previous work with this model has shown that microzooplankton recycling accounts for most of micronutrient recycling fluxes (see Richon et al., 2020); therefore, we focus here on microzooplankton.

### 2.2 Statistical methods to identify recycling drivers

We seek to analyse the biotic and abiotic drivers of recycling and recycling stoichiometry variance. Because of the intimate role of zooplankton recycling in the overall ocean biogeochemical cycling, we could not quantify the role of individual drivers using sensitivity tests. Instead, to identify the role of different factors influencing the variance in micronutrient recycling fluxes and stoichiometry, we used statistical models (Richon et al., 2020). The generalized linear models (GLM) given in Equations (1) and (2) relate the micronutrient recycling flux and the recycling stoichiometry to food quality, prey quantity, sea surface temperature and zooplankton biomass (plus an error term, *ε*):

(1) $$\begin{array}{cc} \text{Micronutrient}\;\text{recycling}\;\text{flux} & =\alpha \;\text{food quality}+\beta \;\text{prey}\;\text{quantity}\\ & \;+\gamma \;\text{sea temperature}+\delta \;\text{Zooplankton biomass}+\varepsilon , \end{array}$$

and

(2) $$\begin{array}{cc} \text{Micronutrient}\;\text{recycling}\;\text{stoichiometry} & =\alpha \;\text{food quality}+\beta \;\text{prey quantity}\\ & \;+\gamma \;\text{sea temperature}+\delta \;\text{zooplankton biomass}+\varepsilon . \end{array}$$

Food quality is calculated similarly as Richon et al. (2020) as the micronutrient stoichiometry (M:C) in zooplankton divided by the micronutrient stoichiometry in preys (Food Quality = (M:C)_zoo_/(M:C)_preys_). This term illustrates the stoichiometric relationship between zooplankton and its preys. When food quality >1, zooplankton stoichiometry (i.e. the micronutrient demand for zooplankton) is higher than that of prey. Therefore, zooplankton may not fulfil its physiological demand for micronutrients through its diet. On the other hand, food quality <1 indicates that prey stoichiometry is higher than zooplankton demand and that zooplankton may ingest excess micronutrients.

Prey quantity is the amount of micronutrient available in the preys (phytoplankton and organic particles), it is defined as the sum of micronutrient concentration in each prey type weighted by microzooplankton preference for each prey (Equation 3):

(3) $$\text{Prey quantity}=\sum_{i}P_{i}^{j}\times p_{i}.$$

In Equation (3), $P_{i}^{j}$ represents the concentration of micronutrient *j* in prey *i* and *p_i_* the preference of zooplankton for prey *i*, with *i* either nanophytoplankton, diatoms or organic particles.

These statistical models explore the roles of the biotic and abiotic parameters directly driving recycling changes. The choice of the explanatory variables in Equations (1) and (2) is based on current scientific understanding of each driver's impact on micronutrient recycling as well as the results from Richon et al. (2020; see also the references therein). Food quality (Schmidt & Hutchins, 1999), prey composition and biomass (Anderson & Harvey, 2019; Steinberg & Landry, 2017) and seawater temperature (Richardson, 2008) have all been shown to influence micronutrient recycling by zooplankton. Zooplankton biomass is expected to directly influence micronutrient recycling since more zooplankton would lead to higher total recycling flux.

The *ε* term is the error term. A high error term may signify that some factors influencing micronutrient recycling were omitted in the GLMs. The omitted factors may be the indirect effects of physics (stratification and modification of currents) and changes in external micronutrient sources (e.g. sedimentary Fe, Mn and Co).

We use the R package relaimpo (https://cran.rproject.org/web/packages/relaimpo/index.html) to calculate the percentage of recycling and recycling stoichiometry variance explained by each statistical model in PRESENT and FUTURE periods. Relaimpo also allows us to calculate the fraction of recycling and recycling stoichiometry variance explained by each of the factors.

## 3 RESULTS

In this section, we use the results from our simulations to study the impacts of climate change on micronutrient recycling and recycling stoichiometry and use the statistical models to identify the main regional drivers of recycling.

### 3.1 Climate change impacts on trace element recycling fluxes and stoichiometry

#### 3.1.1 Recycling fluxes

**FIGURE 1:**
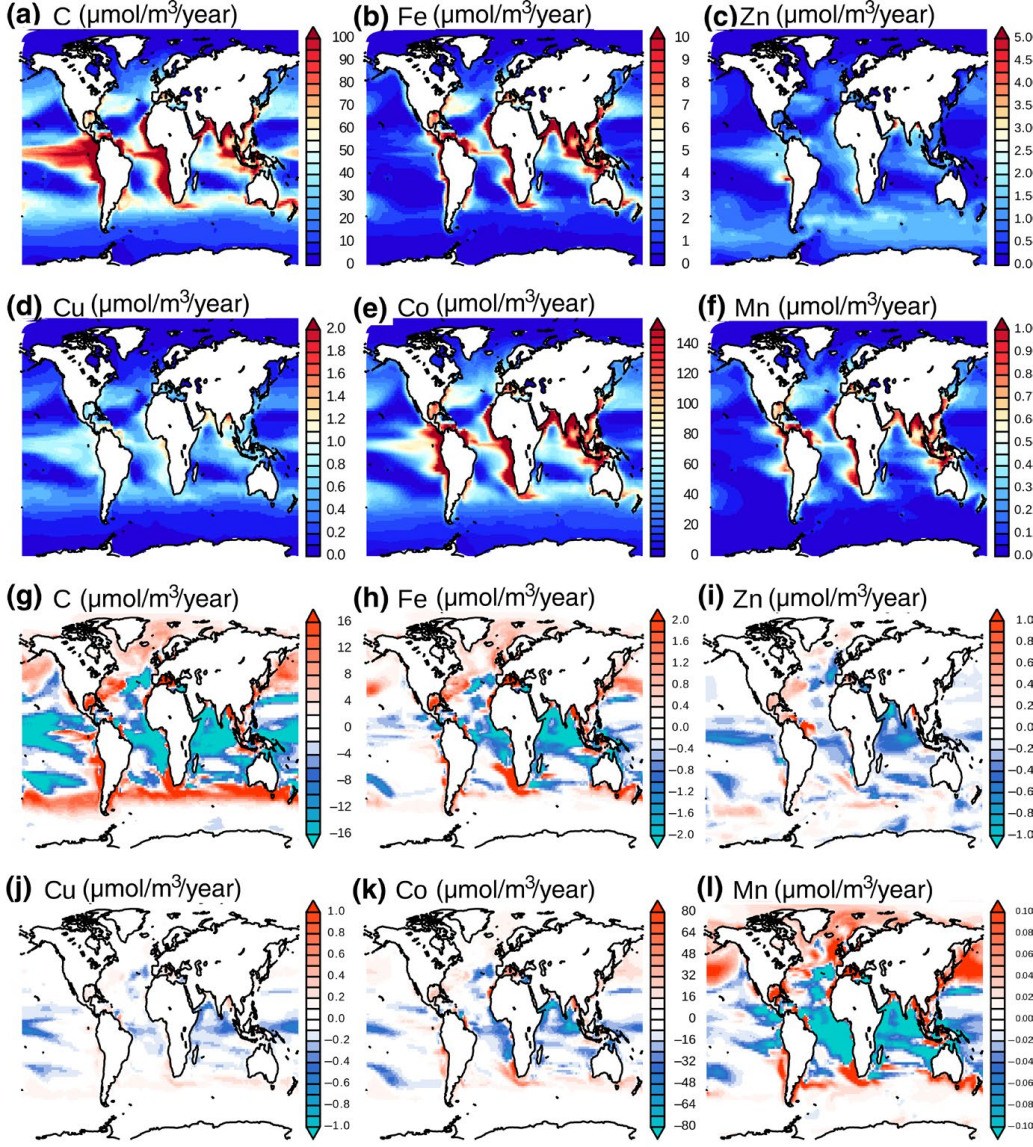
Micronutrient and C recycling fluxes (a–f) averaged in the first 100 m for the PRESENT period. (g–l): changes in microzooplankton trace metal recycling, averaged in the first 100 m, between the 2091–2100 and the 1991–2000 periods

Most microzooplankton recycling of carbon and micronutrients occurs in the most productive coastal and tropical regions as well as in the northern part of the Southern Ocean which host high levels of zooplankton biomass (Figure 1a–f). Micronutrient and C recycling fluxes are well correlated spatially with *R* ^2^ values ranging from 0.95 and 0.97 for Cu and Co to 0.80 and 0.82 for Mn and Fe. These correlations indicate that the regions of high C and micronutrient recycling fluxes are coupled and underpinned by high productivity (correlation between primary productivity and C recycling flux is 0.95 in PRESENT, not shown). Zinc recycling is slightly less well correlated, with an *R* ^2^ of 0.72, largely because it has high rates (around 2 molZn/m^3^/year) in the Southern Ocean.

By 2091–2100, changes in micronutrient recycling fluxes, relative to 1991–2000, are coupled to the overall changes in upper ocean productivity and recycling. There are decreased recycling fluxes in the low‐latitude upwelling regions and increased fluxes in the higher latitudes, such as parts of the Southern Ocean and north Atlantic and Pacific (Figure 1g–l). Projected changes in C recycling mostly reflect the alterations to primary productivity, which shifts towards the high latitudes by the end of the century (Bopp et al., 2013; Tagliabue et al., 2020). This link is illustrated by the *R* ^2^ of 0.94 between spatial changes in primary production and C recycling fluxes. The spatial correlation between changes in C and micronutrient recycling fluxes is high for some micronutrients, such as Cu and Co (*R* ^2^ = 0.87 and 0.78, respectively) and moderate for Fe and Mn (*R* ^2^ = 0.54 and 0.52, respectively). This indicates that the changes in micronutrient recycling fluxes are mostly linked to the changes in primary productivity, which controls C recycling. The main differences between the projected changes in the recycling of Fe and C occur in the Pacific where the model produces a strong decrease in C recycling, but a more muted decrease in Fe (Figure 1g,h). The discrepancies between changes in the recycling fluxes of Mn and C are in the Eastern Tropical Pacific upwelling zone, where C recycling increases by the end of the century, whereas Mn recycling decreases. Also, in the northern part of the Southern Ocean, the projected increase in all micronutrient recycling is weaker than that of C, while the projected increase in Mn recycling is stronger than that of C in the Arctic (Figure 1l). Finally, changes in Zn recycling correlate weakly with the changes in C recycling (*R* ^2^ = 0.28) because Zn recycling fluxes mostly decrease in our model by the end of the century, except for a few regions, like the northern Atlantic sector and some Southern Ocean areas (Figure 1i).

### 3.2 Recycling stoichiometry

**FIGURE 2:**
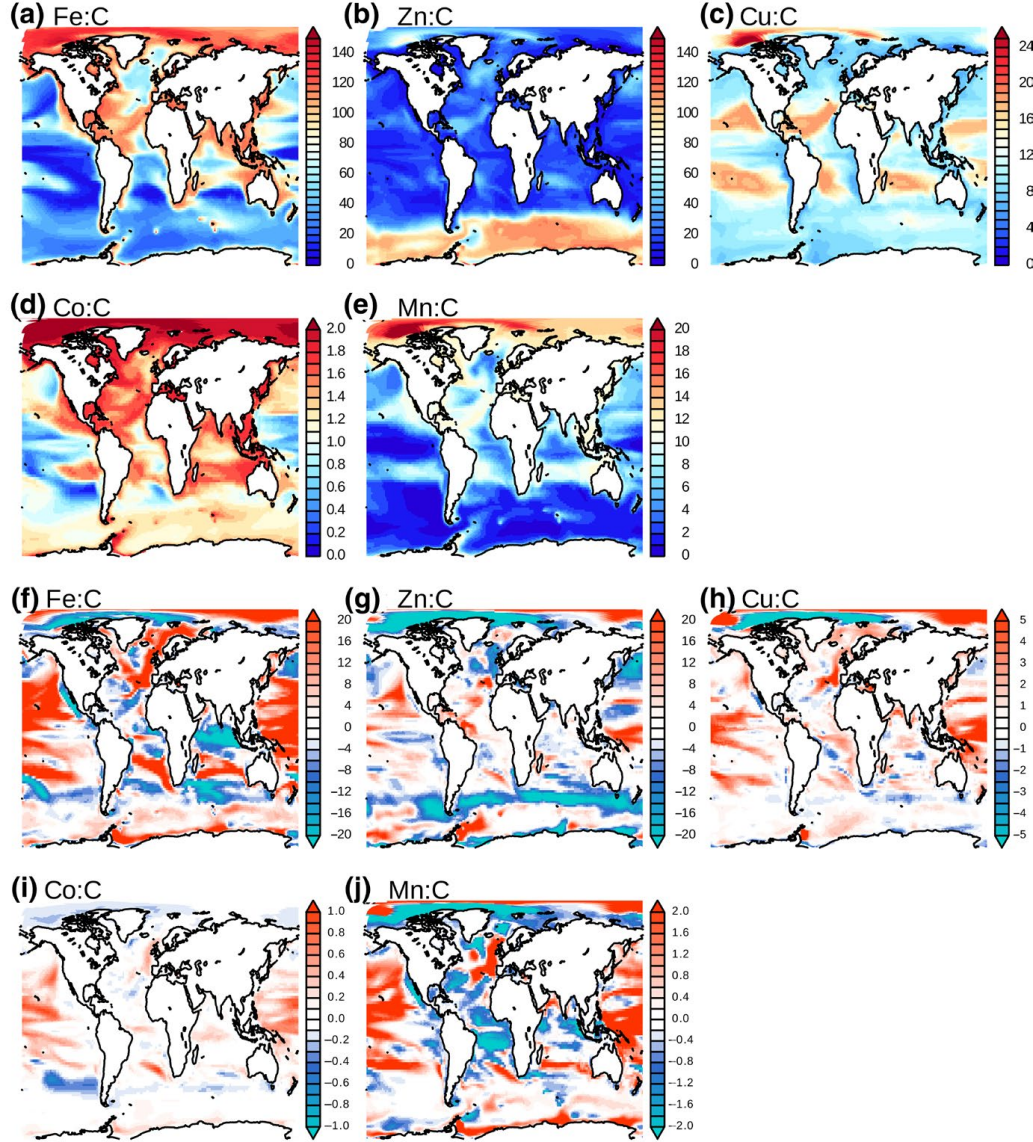
(a–e) Micronutrient recycling stoichiometry in the PRESENT. (f–j) Changes in micronutrient recycling ratio (Micronutrient:C), averaged in the first 100 m, between the 2091–2100 and the 1991–2000 periods. All maps are in mmolMicronutrient:molC, except the Co maps which are in μmolCo:molC

The recycling stoichiometry, relative to C, differs across micronutrients (Figure 2) and is controlled to a large degree by the underlying stoichiometry of the phytoplankton (Figure S3). The spatial correlation between the stoichiometry of recycling and that of phytoplankton for 1991–2000 ranges from 0.91 for Fe and Zn, 0.89 for Mn, 0.88 for Co to 0.68 for Cu. The lower correlation for Cu is driven by the fact that the maximum Cu recycling stoichiometry is located in the Arctic (Figure 2c), whereas the maximum phytoplankton Cu stoichiometry in the model is in the oligotrophic gyres (Figure S3c).

The projected changes in the recycling stoichiometry between 2091–2100 and 1991–2000 are also controlled by the model changes in phytoplankton micronutrient stoichiometry for Fe, Zn and Co (see Figure 2f–j; Figure S4). While the spatial correlations are as high as 0.74 for Fe, 0.86 for Zn and 0.84 for Co, they drop to 0.28 for Cu and 0.11 for Mn. The recycling stoichiometry of all micronutrients is projected to decrease in the northern part of the Southern Ocean, the Arctic and in the upwelling regions of the Eastern Atlantic and the Eastern Tropical Pacific. In the upwelling and Southern Ocean regions, phytoplankton stoichiometry decreases for all micronutrients. In the Arctic, Fe phytoplankton stoichiometry increases by up to 0.6 molFe:molC while Co and Mn phytoplankton stoichiometry remains at saturation by the end of the century. Alternatively, the recycling stoichiometry of micronutrients is projected to increase throughout the oligotrophic gyre regions (in particular in the north Pacific gyre). This response arises because reduced NPP due to enhanced nitrogen limitation (Tagliabue et al., 2020) leads to excess cellular accumulation of micronutrients, which leads to increased phytoplankton micronutrient stoichiometry. For Cu and Mn, changes in the recycling stoichiometry are not clearly linked to parallel changes in phytoplankton micronutrient levels (Figure 2h,i; Figure S4c,e). This may be due to the low maximum cellular quotas imposed for these micronutrients that restricts excess accumulation or the contribution of particulate matter to the zooplankton diet.

### 3.3 Climate‐driven variability in the driving factors of recycling

A detailed description of micronutrient recycling drivers in the PRESENT and the impact of climate change on their distribution is described for these simulations in supplementary material (Text S2; Figures S5 and S6). Here, we focus on assessing how climate change modifies the structural balance between distinct drivers of micronutrient recycling in our model. Using the GLMs described in Section 2.2, we identify the most important factors influencing the variance of micronutrient recycling fluxes and stoichiometry. We chose this approach over performing multiple sensitivity experiments, where each recycling driver is systematically varied, since adjusting the role of zooplankton in our model would lead to wide ranging impacts on ocean biogeochemical cycles that are not easily linked back to our goals. We use ternary plots to visualize the role of the main driving factors for different biogeochemical provinces.

### 3.4 **Drivers of recycling flu**x

**FIGURE 3:**
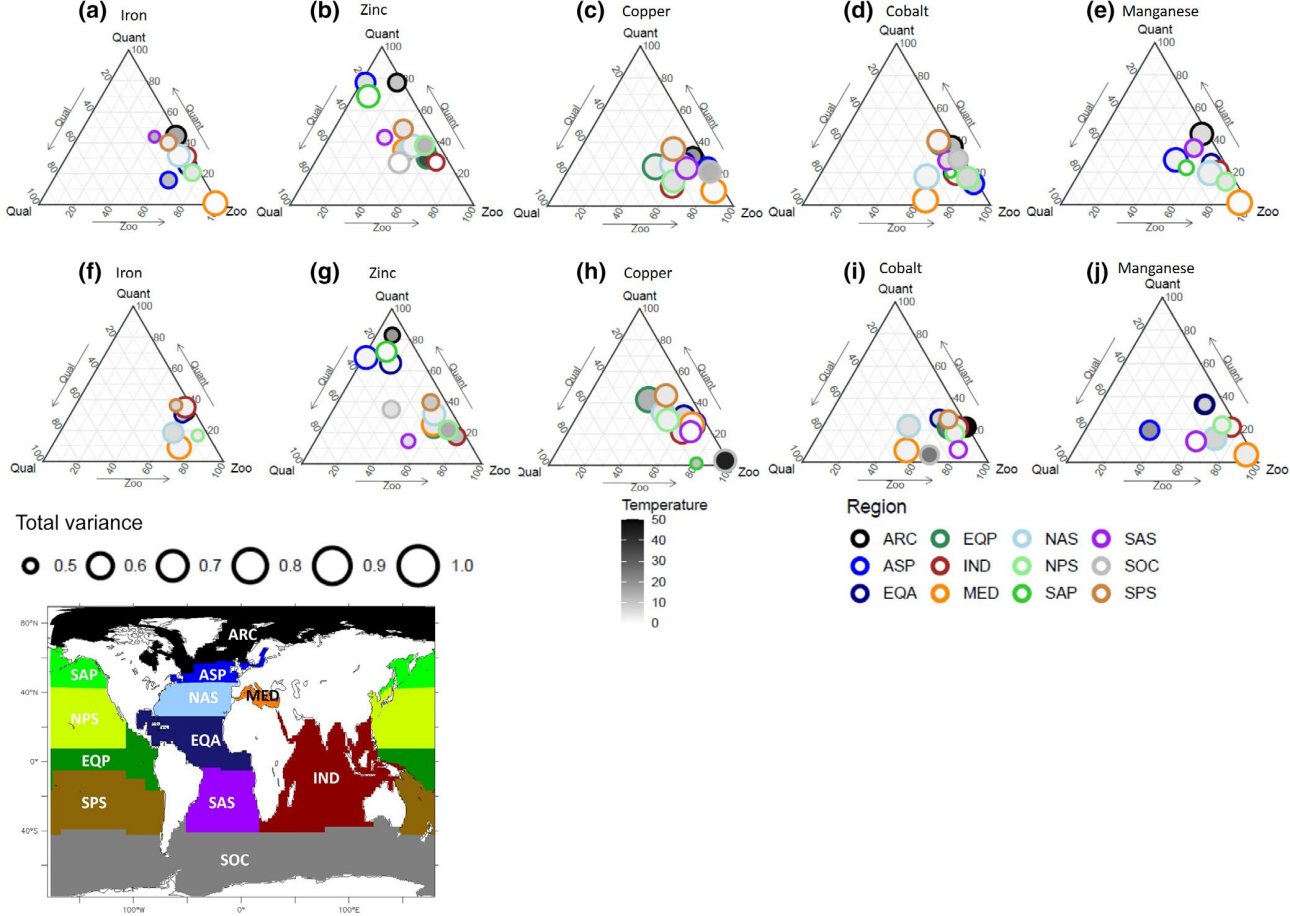
Ternary plots representing the results of the statistical model for micronutrient recycling fluxes in the PRESENT (a–e) and in the FUTURE (f–j). Qual = Food quality, Quant = Prey quantity, Zoo = microzooplankton biomass, circle size indicates the proportion of recycling variance explained by all the variables, outer circle colors represent the different Longhurst regions (regions in which the model explains less than 50% of recycling flux variance are not plotted) the inner color of the circles indicate the proportion of recycling variance explained by temperature

The GLM in Equation (1) explains >50% of micronutrient recycling flux variance for 94% of cases, which means most ocean regions are included in Figure 3. The clustering of Fe, Cu, Co and Mn in the bottom right corner of all Figure 3 ternary plots indicates that zooplankton biomass is the main driver of the variance in their recycling in the PRESENT (1991–2000). The dispersion of some points along the Prey Quantity axis highlights that prey quantity is the secondary driver of the recycling fluxes of these micronutrients. Zn shows a distinct pattern with prey quantity dominating in the Arctic (ARC), south Atlantic sector (SAS) and in the subarctic Atlantic (ASP) and Pacific (SAP) regions (explaining >80% of the Zn recycling variance), while zooplankton biomass is most important elsewhere (Figure 3b). In general, temperature plays a minor role on recycling fluxes in most regions for the PRESENT (inner colours of points in Figure 3), except in the high latitudes such as in the Arctic (ARC), Southern Ocean (SOC) and sub–Arctic Atlantic (ASP) where seasonal changes in temperature explain 10%–20% of micronutrient recycling variance (Figure 3a–e).

The role identified for changes in zooplankton biomass in driving micronutrient recycling variations during the PRESENT continues in the FUTURE period (2091–2100, Figure 3f–j), although other factors become important in some regions and for some micronutrients. For this period, the GLM explains >50% of micronutrient recycling flux variance in 85% of cases. Comparing the upper and lower panels of Figure 3 demonstrates how the driving factors are altered by climate change and illustrates that while zooplankton biomass remains the dominant driving factor of micronutrient recycling flux variance in most regions, the role for sea surface temperature increases, especially in Polar and Pacific regions (Figure 3f–j). Sea surface temperature plays a direct role in driving biological rates in our model (especially in polar systems), but it also covaries with changes to ocean physics that modify nutrient delivery. The role played by prey quantity in driving the variance in recycling fluxes increases for Zn and Cu (Figure 3g,h), while for the subarctic Pacific (ASP), food quality becomes the major driver for Mn (Figure 3j). Regions such as the Equatorial Pacific (EQP), the subarctic regions and the Southern Ocean are not well represented by our statistical model for the FUTURE for Fe and Mn (Figure 3f,j), indicating that the projected changes in Fe and Mn recycling fluxes may be driven by other factors in these regions. Thus, our results indicate that there is the potential for the drivers of micronutrient recycling to be modified by future climate change.

### 3.5 Drivers of recycling stoichiometry

**FIGURE 4:**
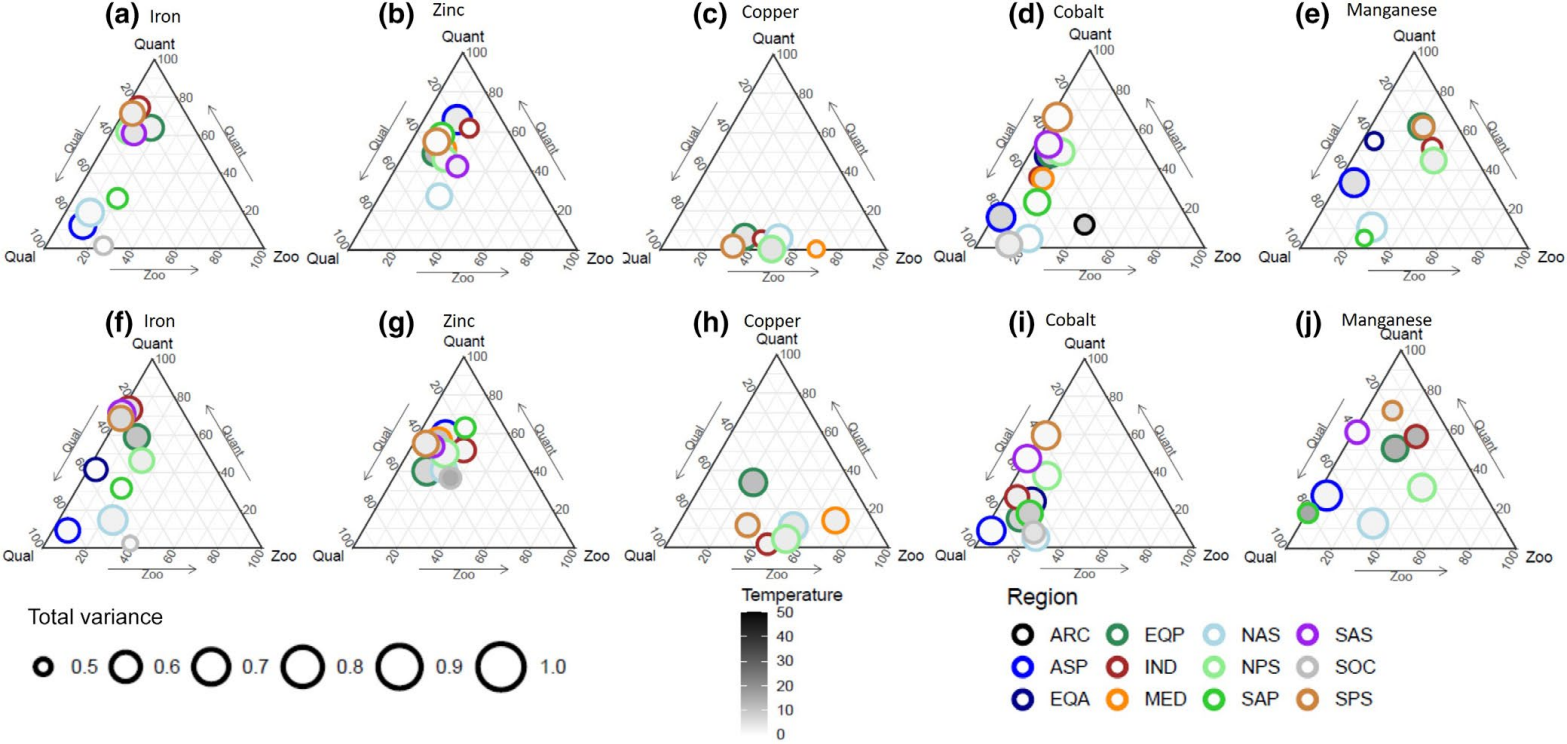
Same as Figure 3 but for recycling ratios (micronutrient:C)

Unlike recycling fluxes, which are mostly driven by zooplankton and prey biomass variability, the drivers of micronutrient recycling stoichiometry are more complex, with different factors emerging depending on the nutrient and the region considered (Figure 4). The GLM in Equation (2) explains >50% of micronutrient recycling flux stoichiometry variance in 75% of cases in the PRESENT (Figure 4a–e). For Fe, there are two clear clusters, with food quality being the main driver in the North Atlantic, sub‐Arctic Pacific and Southern Oceans, and prey quantity being dominant elsewhere (Figure 4a). There is less segregation for Zn, with prey quantity typically dominant, alongside a role for zooplankton biomass and food quality (Figure 4b). An outlier for Zn is the North Atlantic subtropical gyre (NAS) where the importance of food quality is higher (explaining alone about 50% of Zn recycling stoichiometry variance). For Cu, food quality dominates and the role of zooplankton biomass emerges only for the Mediterranean (MED) where it explains about 70% of the variance in Cu recycling stoichiometry (Figure 4c). The ternary plot for Co is similar to Fe, with a regional clustering between dominance by food quality and prey quantity (Figure 4d). Finally, Mn displays another distinct regional pattern with zooplankton biomass, food quality and prey quantity all being dominant in different regions (Figure 4e). Some regions are not always represented in the ternary plots for all micronutrients, indicating that the GLM for micronutrient recycling stoichiometry (Equation 2) has a low explanatory power in these regions. This highlights the role of other factors in our model, such as external inputs associated with aerosols, sediments or rivers (e.g. Bundy et al., 2020; Tagliabue et al., 2020). Finally, temperature plays a minor role in driving micronutrient recycling stoichiometry in all cases as indicated by the white colour of all circles.

The picture that emerges regarding the drivers of the recycling stoichiometry in the PRESENT is altered for the FUTURE in response to climate change (Figure 4f–j). Our GLM retains good skill for the FUTURE period, explaining over half the variance in the projected change in recycling stoichiometry in 72% of cases. Comparing the upper and lower panels of Figure 4 illustrates how climate change affects the structural links between the recycling stoichiometry and its drivers. The regional clustering in the PRESENT become less marked for Fe and Co in the FUTURE period (Figure 4f,i), due to the overall increase in the importance of food quality. Regional differences for Zn also become less evident in the FUTURE, leading to the combined control of the variance in Zn recycling stoichiometry by food quality, prey quantity and zooplankton biomass (Figure 4g). The clear control of Cu recycling stoichiometry by food quality in the PRESENT alters in the FUTURE with prey quantity taking on a stronger role (Figure 4h). The response of Mn recycling stoichiometry becomes strongly variable by region, with each factor emerging as a key driver in different regions across the ocean (Figure 4j). The influence of temperature on the recycling stoichiometry of all micronutrients increases in the FUTURE period (Figure 4f–j), in response to both direct and indirect processes as discussed above.

Overall, our analysis highlights how climate change alters the dominant drivers of the recycling flux and stoichiometry by region and by micronutrient by the end of the century. These structural changes to the mechanisms shaping micronutrient cycling are important as they indicate the potential for feedbacks to emerge under a changing climate. The reduction in skill for the GLM for the FUTURE period also likely indicates the emergence of nonlinear interactions among the factors driving the total rate and stoichiometry of recycling of micronutrients. These interactions or feedbacks can have impacts on the response of micronutrient availability to the biota in response to climate change, both in absolute and relative terms.

### 3.6 Recycling feedbacks on surface micronutrient biogeochemistry

#### 3.6.1 General response of recycling feedbacks

The recycling of micronutrients impacts biogeochemical cycling in two main ways: (1) the gross flux of micronutrients recycled by zooplankton modifies the surface micronutrient inventories and (2) the stoichiometry of recycling impacts the relative availability of dissolved micronutrients, which then affects phytoplankton resource deficiency and limitation (Moore, 2016).

**FIGURE 5:**
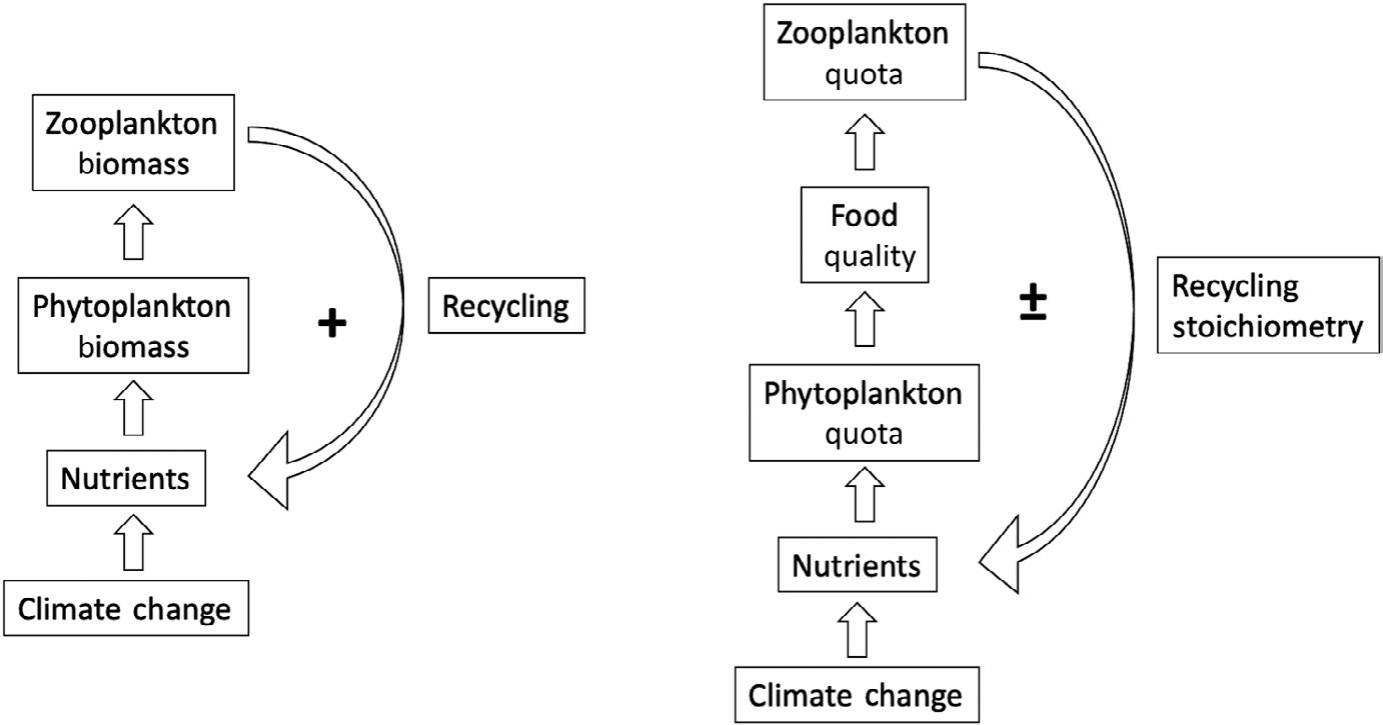
Schematics of the feedbacks of recycling on surface micronutrient biogeochemistry

Figure 5 illustrates the feedbacks of climate change on upper ocean recycling fluxes and stoichiometry. Section 3.2 indicates that the climate‐driven changes in micronutrient recycling fluxes in our model are mostly linked with changes in plankton biomass, which are themselves linked with changes in nutrient concentrations modified by climate change. The declines in zooplankton biomass, projected for most of the low‐latitude oceans due to reduced nutrient availability and NPP (Figure S6; Kwiatkowski, Aumont, & Bopp, 2018; Kwiatkowski, Aumont, Bopp, & Ciais, 2018), are coupled to decreasing micronutrient recycling fluxes (Figure 1), which would deplete upper ocean micronutrient levels still further. Conversely, in the regions where nutrient supply is enhanced and zooplankton biomass is projected to increase, results from PISCES‐BYONIC and the GLMs suggest that gross recycling fluxes will be enhanced, further increasing nutrient availability and upper ocean micronutrient residence times (Figure 1; Figure S6; Sterner, 2009; Tagliabue et al., 2019). Thus, the projected alterations to gross recycling fluxes act to amplify the climate‐driven impacts on overall zooplankton biomass as a positive feedback (Figure 5).

The feedbacks from changes in micronutrient recycling stoichiometry on surface biogeochemistry are more complex since they are strongly influenced by zooplankton food quality in many regions (see Figure 4), which itself depends on the micronutrient stoichiometry of phytoplankton (Figure 2; Figure S4). The differential recycling of resources will change the micronutrient environment of phytoplankton and potentially modify nutrient limitation patterns or intensities in the future (Moore, 2016; Sterner, 2009). The resulting alteration of phytoplankton stoichiometry will feedback on zooplankton food quality (Figure 5). It is important to note that the change in phytoplankton stoichiometry in response to changing recycling stoichiometry is complex and also depends on how far phytoplankton are from their assumed maximum quota and the specific micronutrient uptake kinetics (see Text S1; Table S3), which regulates the specific response of micronutrient uptake rates. Therefore, identifying the general sign of the recycling stoichiometry feedback is complex as it ultimately depends on how food quality deviates from 1 in response to the climate change alterations to recycling stoichiometry (since the zooplankton micronutrient assimilation efficiency increases as food quality gets closer to 1). For example, in the Equatorial Pacific and the Arctic, Zn food quality is projected to increase in response to climate change (see Text S2; Figure S6). The ensuing decline in the Zn recycling stoichiometry then acts to reduce the phytoplankton stoichiometry. This change in phytoplankton stoichiometry then exerts a negative feedback on the recycling stoichiometry that may contribute to lowering Zn food quality. On the other hand, food quality deviations away from 1 are common across many micronutrients (Figures S5 and S6). The resulting increase in the recycling stoichiometry then exerts a positive feedback as the elevated phytoplankton stoichiometry that results drives food quality further still from 1.

#### 3.6.2 Quantifying the impact of food quality feedback on biogeochemistry

**FIGURE 6:**
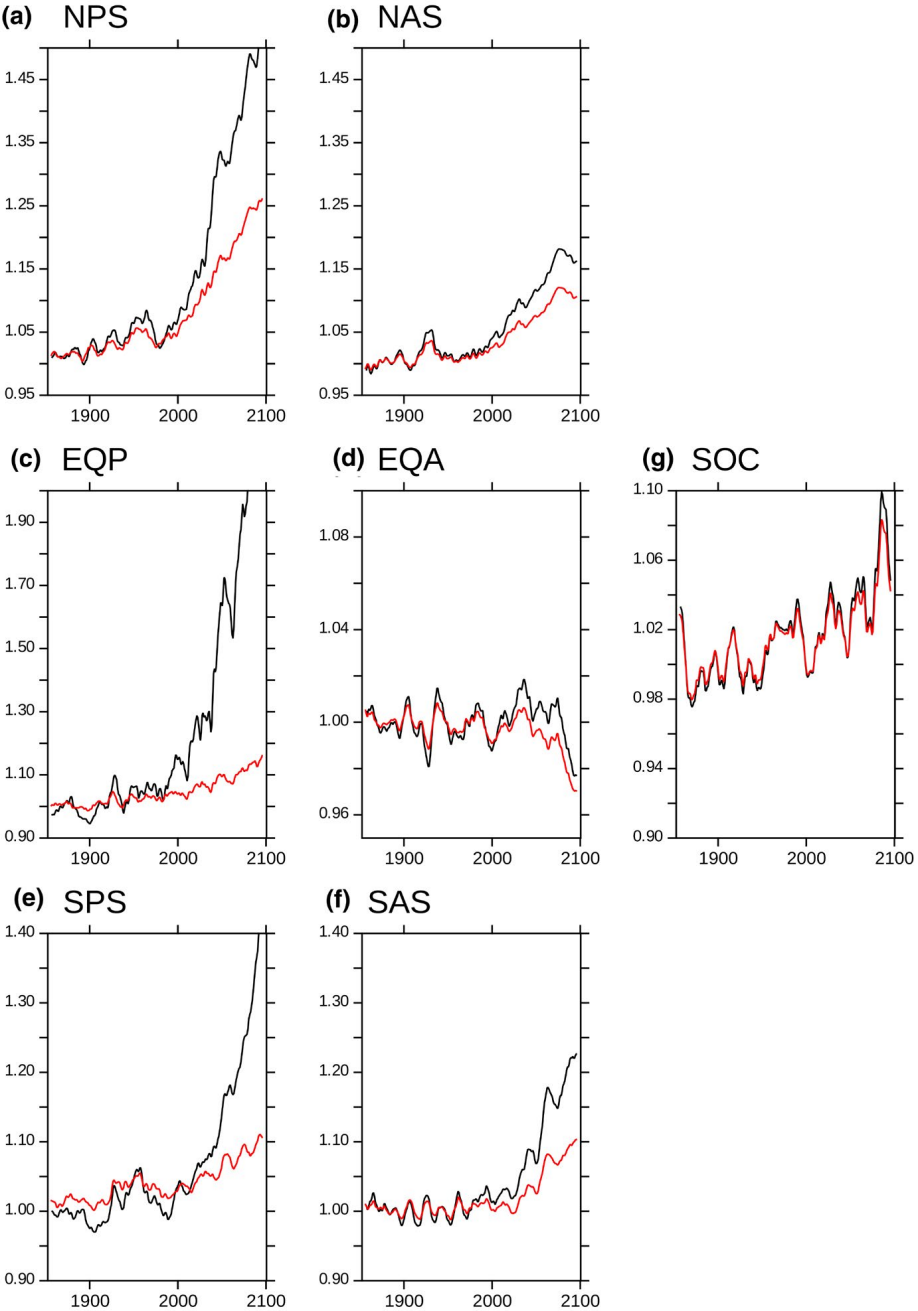
Times series of the trends in iron recycling stoichiometry (normalised against the PICONTROL: 1801–1900 average) in the surface waters of different regions (see Figure 3 for region location). Black lines represent the reference simulation, red lines represent the simulation with fixed food quality

To further explore the nature of the feedbacks associated with the recycling stoichiometry and food quality, we repeated our climate change simulations with food quality factors for micronutrient recycling fixed at their preindustrial levels. In these simulations, the impacts of changes in food quality on micronutrient recycling are eliminated and the recycling stoichiometry only varies in response to changes in zooplankton biomass, prey quantity and temperature. We focus here on the essential micronutrient Fe and examine the trends in the recycling stoichiometry (ratio of the Fe and C recycling rates) normalized to the first hundred years of the PI control simulation (1801–1900) both in the reference simulation and in the simulation with fixed food quality across different biogeochemical provinces (Figure 6).

The recycling stoichiometry of Fe is lower throughout the 21st century when the food quality is fixed (Figure 6). These results highlight how changes in the zooplankton food quality lead to high relative rates of iron recycling in response to climate change, amplifying climate change impacts on Fe recycling stoichiometry. The large differences between the two simulations in the Pacific regions (and to a lesser extent, in the Atlantic) confirm the major role for food quality in driving Fe recycling stoichiometry in those regions (see Figure 4). Variations in Fe food quality in the Atlantic and Southern Ocean are more limited (Figure S6), leading to more muted food quality feedbacks in these regions (Figure 6b,d,f,g).

Both our model and available observations indicate that the low‐latitude Pacific is iron limited. Therefore, recycling changes may trigger important changes in net primary production. Our results indicate that recycling stoichiometry feedbacks lead to greater relative rates of iron recycling due to alterations to the food quality of zooplankton. This enhanced surface retention of iron will contribute to lessening the extent of iron limitation of primary producers in the region, with implications for net primary production projections (e.g. Tagliabue et al., 2020). These amplifying feedbacks illustrated for Fe will likely also be important for Mn and Zn (Figure 2), which also show large projected changes in food quality in the Pacific (Figure S6).

## 4 DISCUSSION

### 4.1 Constraining micronutrient recycling drivers

This article presents results from novel biogeochemical models of micronutrients (see Text S1 for the complete model description). The dissolved micronutrient concentrations are well reproduced by our model in general (see Figures S1 and S2; Table S6), which raises confidence in our projections. However, micronutrient prey content and zooplankton micronutrient food quality are much more complex and available in situ estimates would provide important additional constraints on our model. The phytoplankton and zooplankton micronutrients stoichiometries we apply in our model, which also underlie the calculations of food quality and prey contents, are based on the available measurements from phytoplankton (Twining & Baines, 2013) and zooplankton (Baines et al., 2016; Ratnarajah et al., 2014).

The unusually high complexity of the PISCES‐BYONIC model configuration allows the study of the multiple drivers of micronutrient recycling in an Earth System Modelling framework for the first time. Other global biogeochemical models components of Earth System Models, such as TOPAZ (Dunne et al., 2010) or COBALT (Stock et al., 2014) have similar formulations for Fe plankton stoichiometry and recycling to PISCES, and could be used to enhance our understanding of the uncertainty associated with the impacts of climate change on micronutrient recycling. More broadly, such an effort would help constrain the likely variation in zooplankton food quality across ocean regions. Moreover, eco‐biogeochemical models such as DARWIN (Follows & Dutkiewicz, 2011) may bring complementary information on the impacts of climate change and food quality on microbial diversity. However, it is notable that no other Earth System Model represents any micronutrient other than Fe. Evidence regarding the importance of multiple micronutrients for microbial activity is expanding (Browning et al., 2017, 2021; Peers et al., 2005; Saito et al., 2008; Wu et al., 2019). This highlights the need for a broader consideration of nutrient limitation, recycling and its sensitivity to climate change in Earth System Models.

### 4.2 Implications for ocean biogeochemical cycles

Ocean biogeochemical cycles facilitate the ocean's regulatory role associated with a suite of ecosystem services and are largely governed by dissolved resource concentrations and lower trophic‐level functioning (Bindoff et al., 2019). Modifications to environmental conditions through climate change will impact the drivers of ocean biogeochemical cycles, ranging from changes to nutrient availability to species biomass and physiology (e.g. Kwiatkowski et al., 2020; Laufkötter et al., 2015; Lotze et al., 2019; Tagliabue et al., 2020). Ultimately, the projections of how climate change affects ocean ecosystem functioning are limited by our understanding of the many complex interactions and feedbacks between different biogeochemical drivers. Our results highlight how climate change may lead to multifaceted impacts on the recycling of essential micronutrients, which may, in turn, trigger feedbacks on surface biogeochemistry that can amplify or attenuate the direct impacts of climate change on nutrient and plankton distributions, with implications for marine ecosystems. In this way, projected changes in the fluxes and stoichiometry of microzooplankton recycling themselves affect surface micronutrient concentrations, impacting resource deficiency and ecosystem function. In our model, only the effect of Fe limitation of phytoplankton growth is included at present; therefore, the full suite of impacts across all essential micronutrients is not yet fully accounted for.

Food quality emerged as an important driver of the projected changes in micronutrient recycling dynamics during our experiments. This term depends both on prey, which can vary, and zooplankton stoichiometry, which are currently fixed in our model. Similar to phytoplankton, it is possible that the micronutrient composition of zooplankton may change depending on the species, the size and life stage of the zooplankton, as well as environmental conditions (Baines et al., 2016). Any such variability in the zooplankton micronutrient stoichiometry would then become an additional factor governing the impact of climate change on micronutrient recycling. Kwiatkowski, Aumont, & Bopp (2018) and Kwiatkowski, Aumont, Bopp, & Ciais (2018) showed that accounting for variability in phytoplankton C:N:P quota under the climate change RCP8.5 scenario leads to similar declines in NPP as with a standard fixed stoichiometry version. However, food quality (here phytoplankton N and P content) also varies significantly with declines of 1%–6% globally, but as high as 20% in the Arctic. Because macronutrient and micronutrient recycling are decoupled in PISCES, assumptions regarding N and P stoichiometry do not affect micronutrient recycling. However, the changes in N and P food quality may lead to significant changes in N and P recycling, potentially leading to similar feedbacks than for micronutrients identified in this study.

Sea surface temperature changes are a fundamental component of the changes in ocean biogeochemistry in general because it underpins multiple direct and indirect biotic and abiotic processes (Doney, 2013). In our model, temperature directly affects growth, grazing and recycling rates via metabolic functions (Aumont et al., 2015). However, our model does not account for any potential adaptation responses of the ecosystem to temperature change (Dam, 2012). These adaptive responses are uncertain and difficult to forecast at present but may be important and require further experimental and observational constraints.

### 4.3 Implications for wider ecosystem functioning

The PISCES model represents the lower trophic levels of the planktonic ecosystem and neglects the interaction with zooplankton predators (fish, birds and mammals). However, the effects of temperature and biomass changes may impact zooplankton consumption by higher trophic‐level predators in multiple ways. Shifts in the distribution of zooplankton predators due to shifts in thermal niches and invasion of new potential predators (Cheung et al., 2009) will combine with biomass changes that may modify the balance between zooplankton and predator biomass, with potential consequences for birds or fish stocks (Beaugrand, 2004; Bertram et al., 2001). Combined with the direct influence of temperature changes on the physiology and grazing rates of zooplankton predators, these impacts may further amplify the changes in zooplankton biomass simulated by our model and lead to additional feedbacks on ocean biogeochemistry.

Modifications to food quality and surface ocean stoichiometry may have consequences for upper trophic‐level biomass and nutrient content, which may, in turn, affect human nutrition when commercially important fish are impacted (Hicks et al., 2019; Tagliabue et al., 2020). The broader consequences of changes in microzooplankton recycling we focus on here may have far‐reaching consequences for ocean biogeochemical cycles and ecosystem functioning and require further experimental, field and modelling efforts in the future.

## 5 CONCLUSION

Recycling by microzooplankton occupies a central role in the biogeochemistry of the surface ocean and supports marine ecosystems. In this study, we examined how recycling of micronutrients responds to the change in climate associated with the high emissions RCP8.5 scenario. We find that the recycling of essential micronutrients is impacted by a suite of biotic and abiotic factors, which are all influenced by climate change. Our model experiments show that the net impact of climate change on surface micronutrient recycling results from complex interactions between different drivers, which may affect the rates of stoichiometry of recycling in different directions. As plankton biomass and physiology responds nonlinearly to climate change, the response of upper ocean biogeochemistry may conceal complex multifactorial interactions.

Previous studies examining the impact of climate change on ocean biogeochemical cycles and ecosystem functioning have tended to focus on the direct impacts for nutrient and plankton distributions (Bopp et al., 2013; Kwiatkowski et al., 2020; Laufkötter et al., 2015). However, ocean biogeochemical cycles and ecosystem functioning are determined by a network of complex interactions and feedbacks that include grazers. We find that the projected changes in micronutrient zooplankton recycling dynamics depend on drivers that vary across different micronutrients and by region. Moreover, these drivers often interact and exhibit feedbacks in response to climate change. Therefore, the response of micronutrient recycling by the end of the century is not straightforward. We find that modifications to the magnitude and stoichiometry of recycling lead to feedbacks on biogeochemical cycles. There is a positive feedback between climate change, nutrient levels, zooplankton biomass and gross recycling rates, but the nature of the feedback is much more complex for the stoichiometry of recycling which regulates resource deficiency. Therefore, it is important to understand and quantify the multifaceted drivers of micronutrient recycling by zooplankton and how these impact surface ocean biogeochemistry to reduce uncertainties in the projections of future ocean biogeochemistry.

## Supporting information

Fig S1

Fig S2

Fig S3

Fig S5

Fig S6

Supplementary Material

## Data Availability

Model data is available on Zenodo: https://doi.org/10.5281/zenodo.4723339.
