# Supplementary material for: Biogeochemical feedbacks associated with the response of micronutrient recycling by zooplankton to climate change
Source: Glob Chang Biol. 2021 Jul 29;27(19):4758–70. doi: 10.1111/gcb.15789 (PMC9292334; doi:10.1111/gcb.15789)
Supplement: Supplementary file 1 — Fig S1 [file GCB-27-4758-s003.pdf]

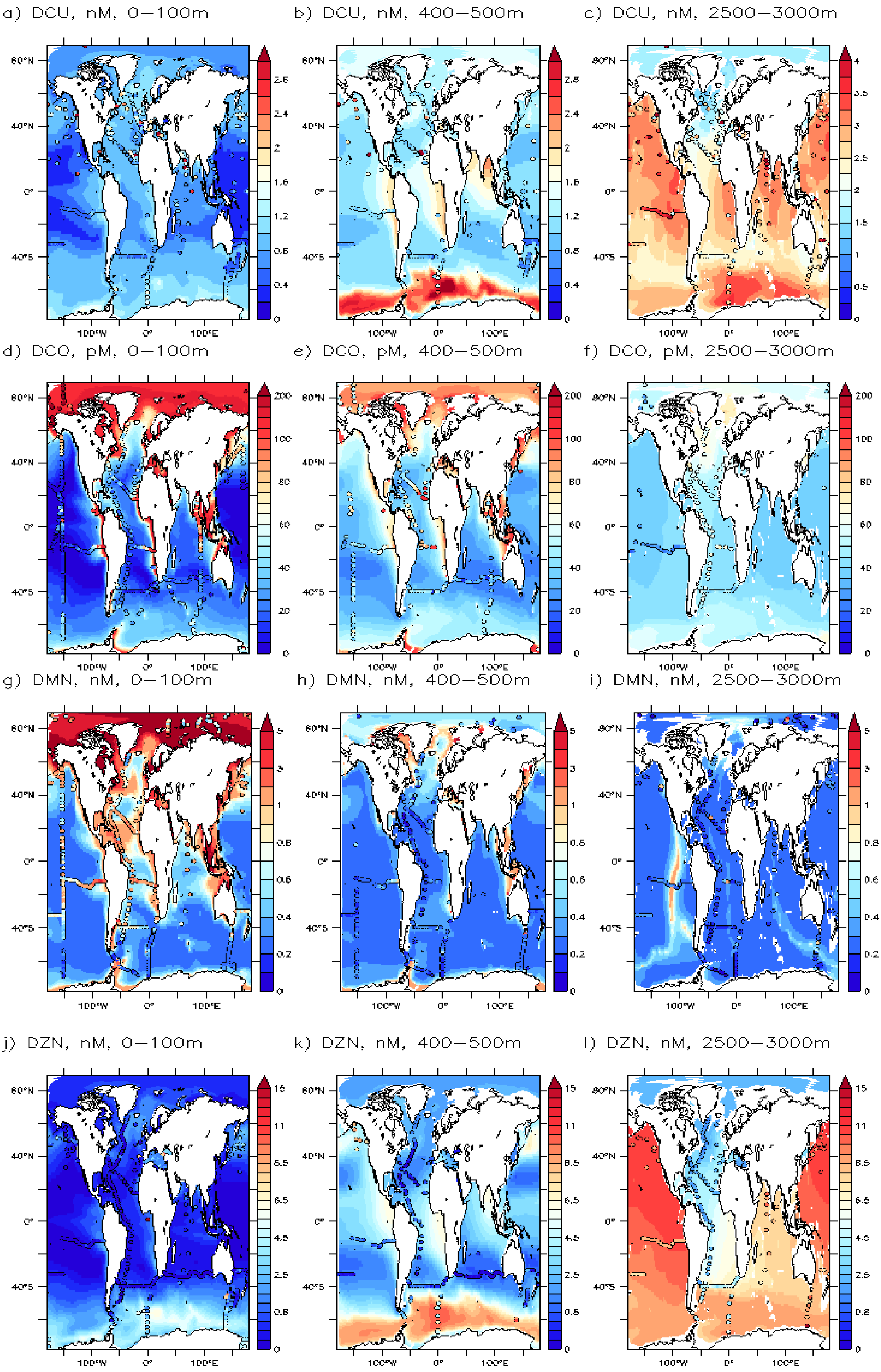
a) DCU, nM, 0–100m
b) DCU, nM, 400–500m
c) DCU, nM, 2500–3000m
d) DCO, pM, 0–100m
e) DCO, pM, 400–500m
f) DCO, pM, 2500–3000m
g) DMN, nM, 0–100m
h) DMN, nM, 400–500m
i) DMN, nM, 2500–3000m
j) DZN, nM, 0–100m
k) DZN, nM, 400–500m
l) DZN, nM, 2500–3000m
60°N
40°N
0°
40°S
100°W
0°
100°E
