# Supplementary figures and images for: Biogeochemical feedbacks associated with the response of micronutrient recycling by zooplankton to climate change
Source: Glob Chang Biol. 2021 Jul 29;27(19):4758–70. doi: 10.1111/gcb.15789 (PMC9292334; doi:10.1111/gcb.15789)

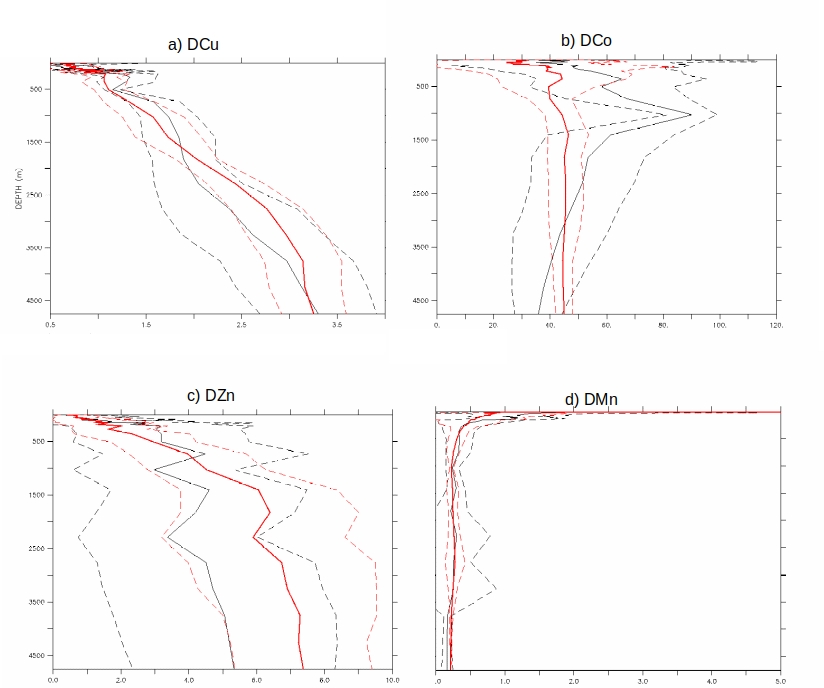

Supplement: Supplementary file 2 — Fig S2 [file GCB-27-4758-s001.jpg]

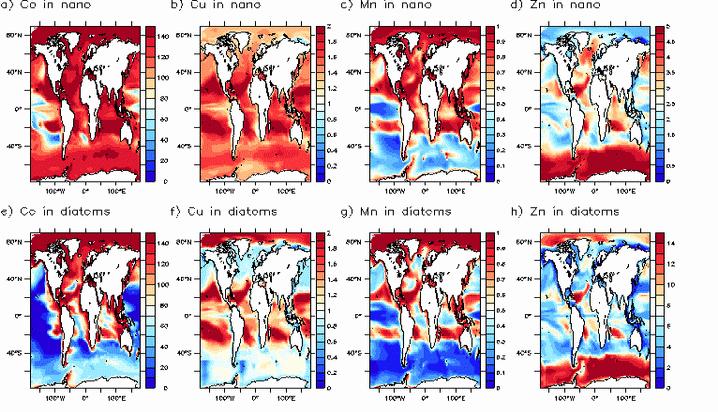

Supplement: Supplementary file 3 — Fig S3 [file GCB-27-4758-s004.jpg]

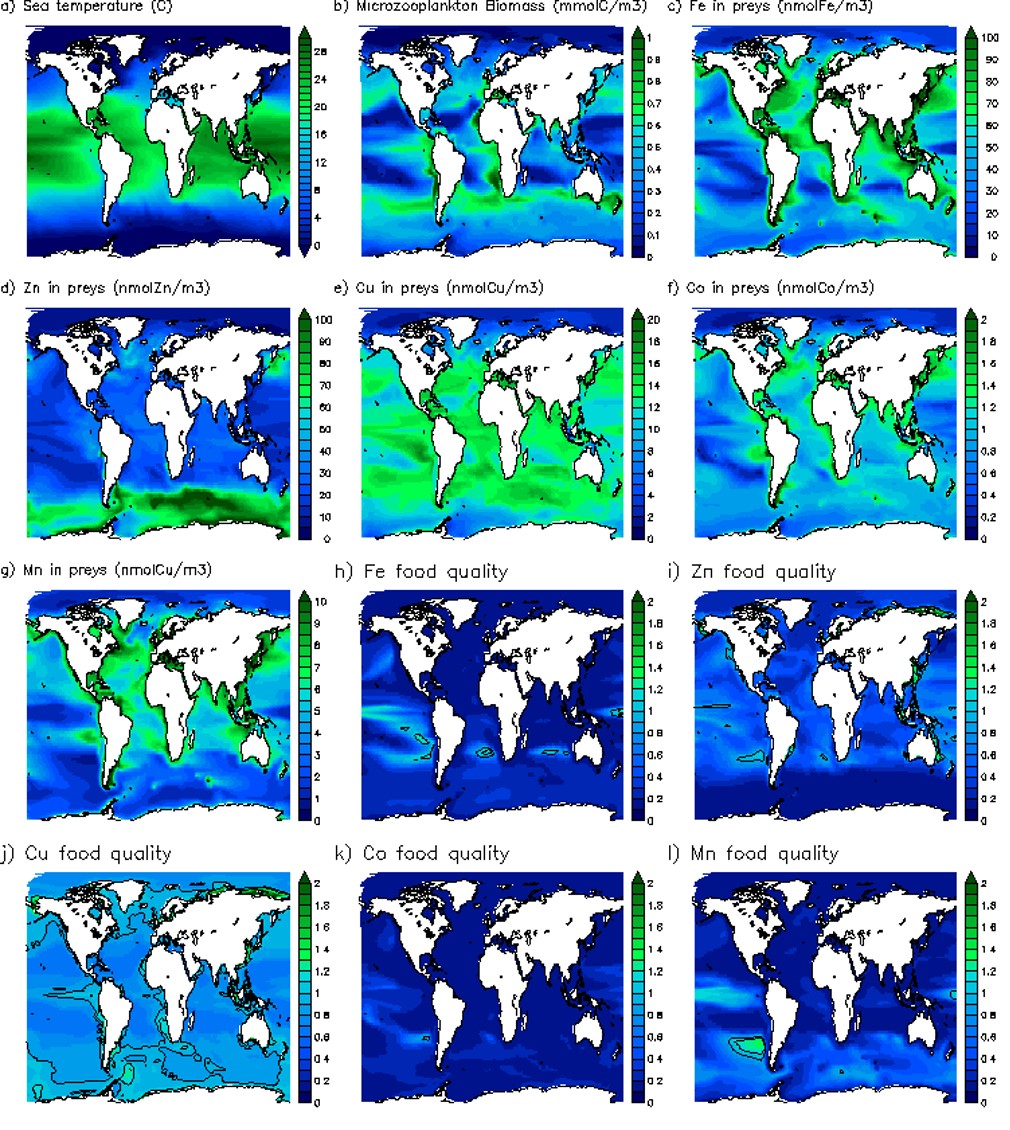

Supplement: Supplementary file 4 — Fig S5 [file GCB-27-4758-s002.jpg]

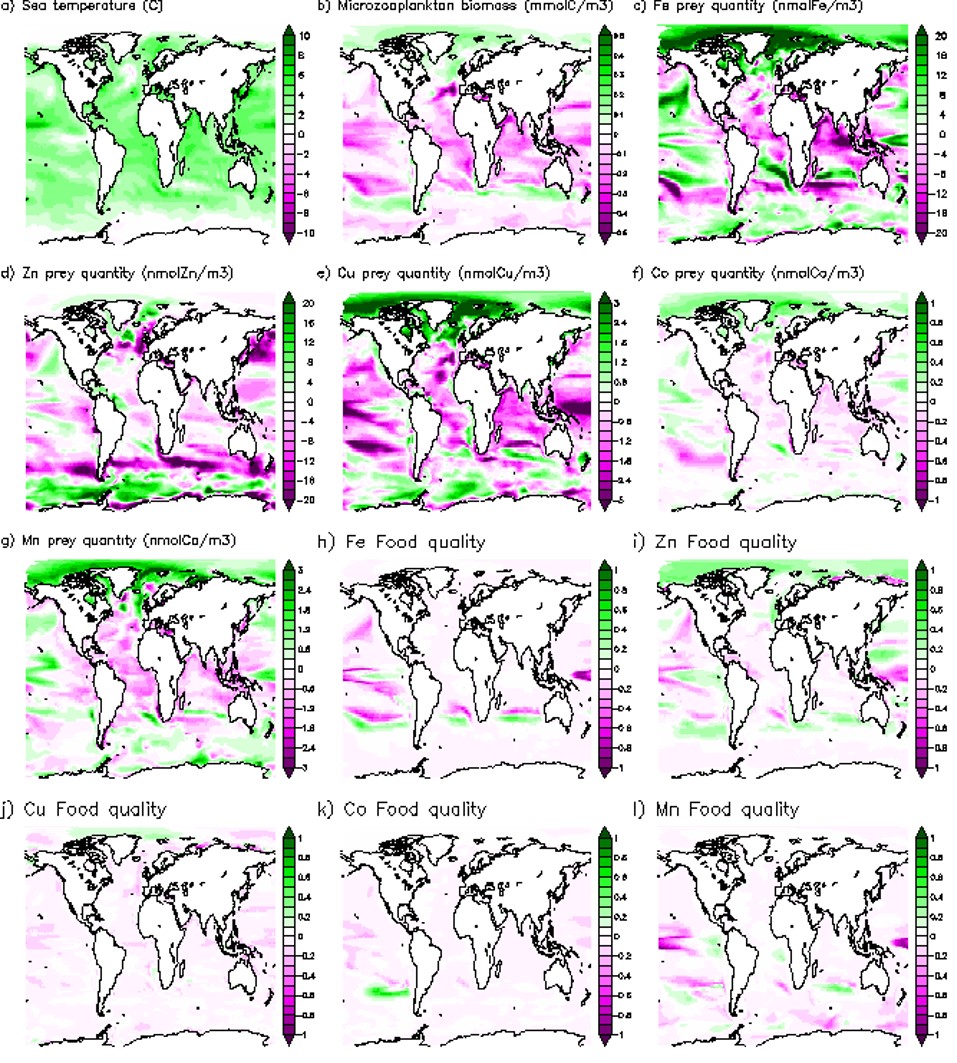

Supplement: Supplementary file 5 — Fig S6 [file GCB-27-4758-s005.jpg]
