# Supplementary material for: Biogeochemical feedbacks associated with the response of micronutrient recycling by zooplankton to climate change
Source: Glob Chang Biol. 2021 Jul 29;27(19):4758–70. doi: 10.1111/gcb.15789 (PMC9292334; doi:10.1111/gcb.15789)
Supplement: Supplementary file 6 — Supplementary Material [file GCB-27-4758-s006.pdf]

Supplementary material for ”Biogeochemical feedbacks associated with the response of micronutrient recycling by zooplankton to climate change”

Camille Richon and Alessandro Tagliabue

S1 Description of the PISCES-BYONIC model

S1.1 Model description

3-D models of Co, Cu, Mn and Zn have been added to the widely used ocean biogeochemical model PISCES [Aumont et al., 2015, Richon and Tagliabue, 2019]. In its default version, PISCES has 24 compartments including 5 limiting nutrients, 2 phytoplankton PFTs (nanophytoplankton and diatoms), 2 zooplankton size classes (microzooplankton and mesozooplankton), oxygen, dissolved inorganic carbon, total alkalinity, calcium carbonate, biogenic silica, dissolved organic carbon and two size classes of sinking particulate organic matter. To add the cycling of the micronutrients Co, Cu, Mn and Zn to PISCES requires 6 new tracers for Co and Mn, 7 for Cu and 9 for Zn. The nomenclature within the code and description of the new tracers are presented in table S1.

S1.2 External sources of micronutrients

Micronutrients are delivered to the ocean via various external sources. River discharge and natural dust are common source for all micronutrients (aerosol Cu also includes anthropogenic sources), Co and Mn have sedimentary sources and only Mn has a hydrothermal source which is scaled on hydrothermal Fe sources (see Table S2).

Sedimentary sources of Co and Mn both occur in shallow coastal areas (below 500m). Sedimentary fluxes of Co and Mn are modelled following [Tagliabue et al., 2018] and are increased at low O_2 (below 150 $\mu molO_2/L$) concentrations by a factor 1000 and 10 respectively, with no sedimentary flux of Co in anoxic regions ($<$ 10 $\mu molO_2/L$).

Micronutrients are delivered to the ocean in dissolved forms. Upon entering the ocean from these sources, micronutrients undergo oceanic biogeochemical cycling following the general equations of section S1.3.

Tracer name	Description
DCO	Dissolved cobalt concentration
CON	Cobalt concentration in nanophytoplankton
COD	Cobalt concentration in diatoms
COP	Cobalt concentration in small organic particles
COG	Cobalt concentration in big organic particles
SCO	Scavenged cobalt concentration
DMN	Dissolved manganese concentration
MNN	Manganese concentration in nanophytoplankton
MND	Manganese concentration in diatoms
MNP	Manganese concentration in small organic particles
MNG	Manganese concentration in big organic particles
SMN	Scavenged Manganese concentration
DCU	Dissolved copper concentration
CUN	Copper concentration in nanophytoplankton
CUD	Copper concentration in diatoms
CUP	Copper concentration in small organic particles
CUG	Copper concentration in big organic particles
SCUP	Scavenged copper concentration on small particles
SCUG	Scavenged copper concentration on big particles
DZN	Dissolved zinc concentration
ZNN	Zinc concentration in nanophytoplankton
ZND	Zinc concentration in diatoms
ZNP	Zinc concentration in small organic particles
ZNG	Zinc concentration in big organic particles
ZNF	Non living frustule associated zinc concentration
ZFD	Diatom frustule zinc concentration
SZP	Scavenged zinc concentration on small particles
SZG	Scavenged zinc concentration on big particles

Table S1: Model tracers associated with micronutrients

S1.3 General equations

S1.3.1 Dissolved micronutrient pools

The dissolved concentrations of all micronutrients follow the general source minus sinks equation (Equation 1):

$$\frac{\delta dM}{\delta t} = \sum Sources_M - Up_M^{\phi} - Scav_M + Remin_M + Recycling_M \qquad (1)$$

In equation 1, M is the micronutrient in question (Co, Cu, Mn or Zn) with dM representing the dissolved concentration of micronutrient M (the sum of the free metal ions and the ligand-micronutrient complexes). All micronutrients have specific ligands with assigned concentrations and stability constants

Name	Description (units)	Value	Reference
Riv_{Co}	Co riverine concentration (mol/L)	2.65E-9	[Gaillardet et al., 2014]
Riv_{Cu}	Cu riverine concentration (mol/L)	0.023E-6	[Gaillardet et al., 2014]
Riv_{Mn}	Mn riverine concentration (mol/L)	0.61E-6	[Gaillardet et al., 2014]
Riv_{Zn}	Zn riverine concentration (mol/L)	9.18E-9	[Gaillardet et al., 2014]
Sol_{Co}	Co solubility in dust	0.08	[Mahowald et al., 2018]
Sol_{Cu}	Cu solubility in dust	0.40	[Paytan et al., 2009]
Sol_{Mn}	Mn solubility in dust	0.25	[van Hulten et al., 2018]
Sol_{Zn}	Zn solubility in dust	0.15	[Mahowald et al., 2018]
Hydro_{Mn}	Mn/Fe ratio from hydrothermal sources	0.33	[Resing et al., 2015]

Table S2: Summary of micronutrient sources parameters.

and the free and ligand-complexed pools of each micronutrient are calculated at equilibrium. $Sources_M$ describes the external sources of micronutrients, Up^{ϕ}_M represents phytoplankton uptake of micronutrient and $Scav_M$, $Remin_M$ and $Recycling_M$ represent scavenging, remineralisation and recycling of micronutrients (see next sections for detailed descriptions).

S1.3.2 Micronutrient concentrations in phytoplankton biomass

General equation for micronutrient concentration in phytoplankton biomass is:

$$\frac{\delta \phi^M}{\delta t} = Up^{\phi}_M - Mort^{\phi}_M - Grazing^{\phi}_M \tag{2}$$

ϕ^M is the micronutrient concentration in phytoplankton group ϕ (diatoms and nanophytoplankton in this version of PISCES), $Mort^{\phi}_M$ is the mortality of phytoplankton ϕ and $Grazing^{\phi}_M$ is the grazing of zooplankton on phytoplankton ϕ. Phytoplankton uptake (Up^{ϕ}_M) is described in detail below.

S1.3.3 Particulate micronutrient pools

All tracers have a small and large organic particulate (detrital) pool. The cycling of these tracers is similar to the PISCES general model [Aumont et al., 2015]. The general equation for the two detrital particulate micronutrient pools is:

$$\frac{\delta M_{part}}{\delta t} = Excr_M + Mort_M + Scav_M + Agg_M - Remin_M - Sink_M \tag{3}$$

Equation 3 is taken from the standard PISCES model and includes micronutrient excretion by zooplankton ($Excr_M$), calculated as the fixed unassimilated fraction of micronutrient prey content. $Mort_M$ represents micronutrient fraction of dead plankton. Agg_M represents the aggregation of particles, which is a function of aggregation constants (positively correlated to organic particle concentrations) and micronutrient:C ratio in organic particles. Finally, $Remin_M$

represents bacterial solubilisation and $Sink_M$ represents sinking, which is identical to the standard PISCES model.
One specificity of micronutrients is their ability to scavenge onto inorganic particles. For Co and Mn, scavenging is a net sink of dissolved micronutrient into the scavenged particulate pool. On the other hand, we track Cu and Zn scavenged reversibly onto small and big particles (see section S1.4.2). The general equation for scavenged particulate micronutrient pools is:

$$\frac{\delta sM}{\delta t} = Scav_M - Dissol_M - Sink_{sM} \tag{4}$$

In Equation 4, sM is the concentration of scavenged micronutrient M, $Scav_M$ is the scavenging flux, $Sink_M$ the sinking rate and (where active) $Dissol_M$ is the dissolution of the scavenged pool. Sinking rate is increasing with depth and is similar for all micronutrients. The scavenging process is described completely in section S1.4.2.
For Zn, there is an additional particulate pool: ZNF which is the Zn fraction in non living diatom frustule (see Table S1). The ZNF pool grows through diatom mortality and follows the cycling of biogeonic silica described in [Aumont et al., 2015].

S1.4 Sinks of dissolved micronutrients: uptake and scavenging

S1.4.1 Phytoplankton uptake

Phytoplankton uptake follows a generic form for all micronutrients (Equation 5):

$$Up_M^{\phi} = [\phi] \times \theta_{max}^{\phi,M} \times \mu_{max}^{\phi} \times \frac{bM}{(bM + ks_M^{\phi})} \times \frac{(1 - \theta^{\phi,M}/\theta_{max}^{\phi,M})}{(1.05 - \theta^{\phi,M}/\theta_{max}^{\phi,M})} \tag{5}$$

In equation 5, $[\phi]$ is the carbon biomass of phytoplankton ϕ (ϕ = N or D for nanophytoplankton and diatoms respectively), Up_M^{ϕ} is phytoplankton ϕ uptake of micronutrient M, $\theta^{\phi,M}$ is the micronutrient:P ratio in phytoplankton cells, $\theta_{max}^{\phi,M}$ is the maximum ratio (described in table S3 and Figure S3). bM is the bioavailable micronutrient concentration and ks_M^{ϕ} is the half saturation constant for micronutrient uptake for phytoplankton ϕ (see table S3). Ligand-bound Cu is considered bioavailable following results from [Semeniuk et al., 2015], therefore, all dissolved Cu is bCu. For Co, dCo is bioavailable for nanophytoplankton while diatoms can only take up free Co [Tagliabue et al., 2018]. On the other hand, only the free form of Mn and Zn are bioavailable.
Diatom Co uptake is influenced by interaction with the dissolved zinc concentration [Tagliabue et al., 2018]:

$$Up_{Co}^{D} = Up_{Co}^{D} \times MAX(0.10; 3 \times (1 - \frac{dZn}{dZn + kCoZn}) \tag{6}$$

This can be implemented in two ways, either directly using the modelled dissolved Zn or by deriving Zn from Si:

$$dZn = (0.065 \times (Si * 1E - 6) + 0.183)) \tag{7}$$

In equation 6, $kCoZn$ is the half saturation constant for Co-Zn interaction for diatom uptake (0.5 nmolZn/L) [Tagliabue et al., 2018].
In the model, there is an optional feedback of iron limitation on Zn uptake.

$$Up_{Zn}^{\phi} = Up_{Zn}^{\phi} \times \frac{(4 - 4.5 \times xlim_{Fe}^{\phi})}{(xlim_{Fe}^{\phi} + 0.5)} \tag{8}$$

With $xlim_{Fe}^{\phi}$ the Fe limitation term for phytoplankton ϕ, which tends towards 4 when phytoplankton is Fe-limited and tend towards 1 when Fe concentration is high [Aumont et al., 2015]. This feedback increases Zn uptake as Fe uptake is upregulated under phytoplankton Fe-limitation as observed in [Cullen et al., 2003].
A proportion of the Zn uptake by diatoms is allocated in the frustule (see table S3).

Name	Description (units)	Value	Reference
ks_{Co}	Half saturation constant for Co uptake (molCo/L)	50E-12, 80E-12	[Tagliabue et al., 2018]
$\theta_{max}^{\phi,Co}$	Maximum Co:P ratio in phytoplankton cells (molCo:molP)	150E-6	[Twining and Baines, 2013]
ks_{Cu}	Half saturation constant for Cu uptake (molCu/L)	4E-9, 12E-9	[Richon and Tagliabue, 2019, Guo et al., 2010]
$\theta_{max}^{\phi,Cu}$	Maximum Cu:P ratio in phytoplankton cells (molCu:molP)	2E-3	[Twining and Baines, 2013]
ks_{Mn}	Half saturation constant for Mn uptake (molMn/L)	2E-9, 6E-9	
$\theta_{max}^{\phi,Mn}$	Maximum Mn:P ratio in phytoplankton cells (molMn:molP)	0.976E-3	[Twining and Baines, 2013]
ks_{Zn}	Half saturation constant for Zn uptake (molZn/L)	0.2E-9, 0.6E-9	
$\theta_{max}^{\phi,Zn}$	Maximum Zn:P ratio in phytoplankton cells (molZn:molP)	5E-3, 15E-3	[Twining and Baines, 2013]
znf	Proportion of diatom Zn uptake allocated to the frustule	0.03	

Table S3: Summary of micronutrient limitation terms for phytoplankton. When 2 values are given, the first is for nanophytoplankton, the second for diatoms.

S1.4.2 Scavenging

Scavenging is represented in specific ways for micronutrients.
For Cu and Zn, we assume reversible scavenging represented as the continuous exchange between inorganic Cu' and Zn' and organic particles.

$$Scav_M = \frac{ztrc}{(ztrc + 1) \times (M')} - sM \tag{9}$$

$$ztrc = Part_C \times KD_M \tag{10}$$

In equation 9, M' and sM describe inorganic micronutrient (Cu or Zn) and scavenged micronutrient respectively. In equation 10, $Part_C$ is the concentration of organic particles and KD_M is the partition coefficient (Table S4). When the result of equation 9 is positive, net scavenging is occurring, when this term is negative (i.e. low particles concentration) inorganic micronutrient is released from particles.

Name	Description (units)	Value	Reference
KD_{Cup}	Partition coefficient for Cu scavenging on small particles (1/mmol)	200E-3	[Richon and Tagliabue, 2019]
KD_{Cug}	Partition coefficient for Cu scavenging on big particles (1/mmol)	10E-3	[Richon and Tagliabue, 2019]
KD_{Znp}	Partition coefficient for Zn scavenging on small particles (1/mmol)	30E-3	[Weber et al., 2018]
KD_{Zng}	Partition coefficient for Zn scavenging on big particles (1/mmol)	1E-3	[Weber et al., 2018]

Table S4: Partition coefficients for Cu and Zn scavenging.

Co and Mn scavenging is represented via the precipitation of bacterial Mn oxides that can incorporate Co. Therefore, it is a function of temperature, bacterial biomass, oxygen concentration and light. Greater oxygen, temperature and bacterial biomass increases the scavenging of Mn and Co, whereas high light and low oxygen reduce the Mn and Co scavenging rate. The dissolution of Mn oxides and Co containing Mn oxides is a function of light and low oxygen. The scavenging parameterisation is described in detail by [Tagliabue et al., 2018]. The Q10 values for the base rate are specific for Mn and Co and taken from [Lee and Fisher, 1994].

S1.5 Resupply of micronutrients by remineralization and recycling

S1.5.1 Remineralization

Remineralization describes the process through which dissolved micronutrients are delivered to the water column from particles solublisation by bacteria. Rem-

ineralization rates are identical for all micronutrients and increases with temperature and bacterial biomass as for the base PISCES model [Aumont et al., 2015].

S1.5.2 Recycling

Recycling is the resupply of dissolved micronutrients by zooplankton. It has been described for iron in [Richon et al., 2020] and is modelled identically for all micronutrients.

$$Recycling_M = Grazing_M \times (1 - unass) \times \theta^{\phi,M} - \theta^{zoo,M} \times Feeding_{eff} \quad (11)$$

$Grazing_M$ is the flux of micronutrient ingested by zooplankton by grazing, it is dependant on the zooplankton grazing rate (which varies with temperature) and on micronutrient prey concentration. The unassimilated fraction of the preys ($unass$) is constant at 0.3, $\theta^{zoo,M}$ is the zooplankton micronutrient stoichiometry, which is fixed and specific for each micronutrient (table S5). Finally, $Feeding_{eff}$ is the zooplankton feeding efficiency which depends on the micronutrient food quality.

Name	Description (units)	Value	Reference
$\theta^{zoo,Co}$	Zooplankton Co stoichiometry (μmolCo/molC)	0.16	[Twining and Baines, 2013]
$\theta^{zoo,Cu}$	Zooplankton Cu stoichiometry (μmolCu/molC)	10	[Ratnarajah et al., 2014]
$\theta^{zoo,Mn}$	Zooplankton Mn stoichiometry (μmolMn/molC)	1	[Ratnarajah et al., 2014]
$\theta^{zoo,Zn}$	Zooplankton Co stoichiometry (μmolZn/molC)	10	[Baines et al., 2016]

Table S5: Zooplankton micronutrient quotas

S1.6 Model results

In this section, we aim to provide a comparison of some key model results with available observations. Figure S1 presents the spatial distribution of dissolved micronutrients at different depths compared with observations from the GEOTRACES program [Schlitzer et al., 2018].
Figure S2 represents the globally averaged vertical profiles of micronutrients and Table S6 reports the associated statistics against the GEOTRACES data. The global profiles confirm that the model captures well the vertical distribution of micronutrients. The linear shape of the Cu profile, the hybrid nature of the Co profile, the deep nutrient-like profile of Zn and the scavenged profile of Mn are all well represented by the model (Figure 2). Obvious shortcomings are Co between 700 and 1000 m which seems underestimated by the model (likely due to the mismatch in model oxygen minimum zones in the Atlantic [Tagliabue et al., 2018]) and Mn in surface which seems overestimated (likely due to the contribution of the Arctic ocean).
Spatially, the model also captures the general patterns of micronutrient distributions from the available GEOTRACES observations. The generally low and

uniform surface values of Cu, alongside the regional distinctions and the increase with depth are well captured by the model (Figure 1a-c and [Richon and Tagliabue, 2019]). The the low offshore Co concentrations, high Co levels in the Arctic and in shallow coastal areas, as well as the impact of oxygen minimum zones in the ocean interior are reproduced well by the model (Figure 1d-f and [Tagliabue et al., 2018]). The model captures the regional differences in surface Zn, including the elevated levels in the Southern Ocean and Atlantic - Pacific contrast at depth (Figure 1j-l). Finally, the model is able to reproduce the high concentrations of Mn in the surface Atlantic and in coastal areas, Mn depletion in the Southern Ocean and low deep Mn concentrations (Figure 1g-i). Concentrations of Mn are overestimated by the model in the surface Arctic region and along the Pacific ridge where the hydrothermal signature is overestimated. The model also underestimates spatial variability in DMn in the surface Pacific.

Results reported in Table S6 show that the model generally has excellent skill for Cu and Zn and while the skill is more muted for Co and Mn, it is comparable to other hybrid-type elements and prior studies. The model generally displays poorer performances between 200 and 1000 m, which is the depth range of oxygen minimum zones, which can be particularly problematic in the Atlantic Ocean. For instance, the poorer performances for Cu in this depth range may be explained by the overestimated concentrations in the OMZ (see also [Richon and Tagliabue, 2019] for a discussion), with an opposite response for Co (see also [Tagliabue et al., 2018] for a discussion). It is also important to note that the 200-1000 m depth range has the least data points for all micronutrients and there are many ocean regions without any data coverage. The lower correlation between the model and data for Mn away from the surface may indicate the need for further tuning of the sensitivity to oxygen or the role of sedimentary and deep hydrothermal vent sources. Nevertheless, the model results for Mn are similar to those of [Hulten et al., 2017].

**Depth range**	**Cu**	**Co**	**Mn**	**Zn**
0-100 m	0.52 (898)	0.75 (1750)	0.40 (2098)	0.49 (1648)
100-200 m	0.61 (657)	0.72 (1472)	0.10 (1609)	0.58 (1144)
200-500 m	0.46 (222)	0.65 (535)	0.14 (598)	0.54 (499)
500-1000 m	0.17 (73)	0.51 (153)	0.08 (144)	0.82 (137)
1000-2500 m	0.41 (179)	0.61 (191)	0.44 (403)	0.92 (346)
2500-5000 m	0.55 (370)	0.58 (383)	0.37 (873)	0.94 (733)
Global	0.84 (2168)	0.58 (3844)	0.49 (4957)	0.73 (3921)

Table S6: Regression results between model and data. The numbers represent the R of the regression between model and data, numbers in parenthesis are the number of observations for each depth range.

S2 Climate change impacts on zooplankton recycling drivers

We examined the relative role played by sea surface temperature, zooplankton biomass, prey quantity and prey food quality on micronutrient recycling. In the PRESENT (1991-2000), sea surface temperature (Figure S5a) follows a latitudinal gradient that increases away from the poles, punctuated by drops where colder deep water upwells. Microzooplankton biomass (Figure S5b) is highest (8 to 10 mg/m3) in the most productive regions where the phytoplankton biomass is also high, such as the coastal, tropical, and upwelling regions. Prey quantity (Figure S5c-g) reflects the micronutrient concentration in phytoplankton and organic particles as a function of the grazing preferences. Broadly speaking, the micronutrient prey quantity follows the modelled patterns in phytoplankton biomass, but there are some notable differences between the micronutrients. The prey quantity of Fe, Co and Mn show a similar distribution, with high quantities in the most productive regions. Zn prey quantity shows a marked maximum in the Southern Ocean because of the known high Zn content in southern ocean diatoms (Figure S5d). The prey quantity of Cu is high in most ocean regions, except in the polar regions because of the variations in the phytoplankton Cu content (see Figure S3c). The spatial patterns for food quality show more complex patterns across the range of micronutrients.

The food quality factors for all micronutrients (Figure S5h-l) are inferior to 1 in most ocean regions, indicating that microzooplankton food is generally rich in micronutrients. Cu contrasts with this general pattern and is found in similar proportions in predators and preys in most of the ocean in our model (food quality close to 1, Figure S5j), because the phytoplankton Cu quota shows low spatial variability (see Figure S3). Co and Fe food quality have similar distributions, with a food quality factor generally low, meaning that zooplankton are feeding on prey that are Fe and Co rich (Figure S5h and k). Zn food quality is close to 1 in the low and mid latitude regions, but much lower than 1 in the Southern Ocean due to the high proportion of Zn in phytoplankton (Figure S3). The South Pacific region displays a food quality factor for Mn that exceeds 1, (Figure S5l) because the low phytoplankton Mn levels are unable to match the zooplankton Mn requirement (see Figure S3). The food quality factor can be used to explain the patterns of recycling that occur. According to the findings from [Richon et al., 2020], a food quality factor close to 1 favours low recycling due to an optimal match between nutritional availability and requirements. This can explain the low recycling of Cu throughout the ocean, of Fe, Co and Mn in the equatorial Pacific regions, and of Mn in the Southern Ocean where food quality factors are close to 1 (Figure S5h,j,k,l). Any deviation in the food quality factor from 1 leads to increased recycling because of reduced micronutrient assimilation efficiency in our model. This explains the high Zn recycling in the Southern Ocean, where the food quality factor is very low (Figure S5i). The differences in food quality factors across micronutrients in our

model arise from the biogeochemical processes that drive micronutrient surface distributions, influencing the degree of mismatch between the stoichiometries of phytoplankton and zooplankton. Taken as a whole, our model results suggest that in every oceanic region, zooplankton physiology would be predominantly influenced by different micronutrients in distinct regions. For instance, in the Southern Ocean, zooplankton graze on Fe, Zn and Co rich prey, on prey that are Mn deficient and Cu replete. Alternatively, in the Equatorial Pacific, although food quality is close to 1 for all micronutrients, implying that the zooplankton diet is well-balanced in micronutrients, recycling fluxes are high (Figure 1), highlighting the role played by other factors.

Future climate change impacts on micronutrient recycling dynamics are driven by the combined influence of a range of different direct and indirect drivers. The modelled changes in sea surface temperature and microzooplankton biomass by the end of the century are consistent with previous projections [Kwiatkowski et al., 2020, Bopp et al., 2013], with increased temperature by up to 8 to 10°C and increased biomass by 1 to 2 mg/m3 in the Arctic and the northern part of the Southern Ocean and decreased biomass by up to 3 mg/m3 at lower latitudes (Figure S6a,b). Changes in micronutrient prey quantity reflect the combined impact of changes in prey stoichiometry and biomass and generally increase at higher latitudes and decline at lower latitudes (Figure S6c-g). However, there are some deviations from this general pattern, with Cu prey quantity decreasing by up to 2 nmolCu/m3 in the Pacific gyres where the prey quantity of Fe, Co and Mn is projected to increase, both in response to changes in prey micronutrient stoichiometry. The impacts of climate change on food quality factors (Figure S6h-l) remain low for most regions. For instance, despite the marked changes in recycling and recycling stoichiometry of all micronutrients in the Southern Ocean and in the Atlantic Ocean (Figures 1 and 2), food quality changes are close to 0 for all micronutrients in these regions, indicating that other factors are responsible for the projected changes in recycling. The range of directional changes in the recycling drivers and the diversity of responses for each micronutrient make the future fate of micronutrient recycling difficult to predict.

References

[Aumont et al., 2015] Aumont, O., Ethé, C., Tagliabue, A., Bopp, L., and Gehlen, M. (2015). PISCES-v2: an ocean biogeochemical model for carbon and ecosystem studies. *Geoscientific Model Development*, 8(8):2465–2513.

[Baines et al., 2016] Baines, S. B., Chen, X., Twining, B. S., Fisher, N. S., and Landry, M. R. (2016). Factors affecting Fe and Zn contents of mesozooplankton from the Costa Rica Dome. *Journal of Plankton Research*, 38(2):331–347.

[Bopp et al., 2013] Bopp, L., Resplandy, L., Orr, J. C., Doney, S. C., Dunne, J. P., Gehlen, M., Halloran, P., Heinze, C., Ilyina, T., Séférian, R., Tjiputra, J., and Vichi, M. (2013). Multiple stressors of ocean ecosystems in the 21st century: projections with CMIP5 models. *Biogeosciences*, 10(10):6225–6245.

[Cullen et al., 2003] Cullen, J. T., Chase, Z., Coale, K. H., Fitzwater, S. E., and Sherrell, R. M. (2003). Effect of iron limitation on the cadmium to phosphorus ratio of natural phytoplankton assemblages from the Southern Ocean. *Limnology and Oceanography*, 48(3):1079–1087. _eprint: https://aslopubs.onlinelibrary.wiley.com/doi/pdf/10.4319/lo.2003.48.3.1079.

[Gaillardet et al., 2014] Gaillardet, J., Viers, J., and Dupré, B. (2014). Trace Elements in River Waters. In *Treatise on Geochemistry*, pages 195–235. Elsevier.

[Guo et al., 2010] Guo, J., Annett, A. L., Taylor, R. L., Lapi, S., Ruth, T. J., and Maldonado, M. T. (2010). COPPER-UPTAKE KINETICS OF COASTAL AND OCEANIC DIATOMS1. *Journal of Phycology*, 46(6):1218–1228.

[Hulten et al., 2017] Hulten, M. v., Middag, R., Dutay, J.-C., Baar, H. d., Roy-Barman, M., Gehlen, M., Tagliabue, A., and Sterl, A. (2017). Manganese in the west Atlantic Ocean in the context of the first global ocean circulation model of manganese. *Biogeosciences*, 14(5):1123–1152.

[Kwiatkowski et al., 2020] Kwiatkowski, L., Torres, O., Bopp, L., Aumont, O., Chamberlain, M., Christian, J. R., Dunne, J. P., Gehlen, M., Ilyina, T., John, J. G., Lenton, A., Li, H., Lovenduski, N. S., Orr, J. C., Palmieri, J., Santana-Falcón, Y., Schwinger, J., Séférian, R., Stock, C. A., Tagliabue, A., Takano, Y., Tjiputra, J., Toyama, K., Tsujino, H., Watanabe, M., Yamamoto, A., Yool, A., and Ziehn, T. (2020). Twenty-first century ocean warming, acidification, deoxygenation, and upper-ocean nutrient and primary production decline from CMIP6 model projections. *Biogeosciences*, 17(13):3439–3470. Publisher: Copernicus GmbH.

[Lee and Fisher, 1994] Lee, B.-G. and Fisher, N. (1994). Effects of sinking and zooplankton grazing on the release of elements from planktonic debris. *Marine Ecology Progress Series*, 110:271–281.

[Mahowald et al., 2018] Mahowald, N. M., Hamilton, D. S., Mackey, K. R. M., Moore, J. K., Baker, A. R., Scanza, R. A., and Zhang, Y. (2018). Aerosol trace metal leaching and impacts on marine microorganisms. *Nature Communications*, 9(1):2614.

[Paytan et al., 2009] Paytan, A., Mackey, K. R. M., Chen, Y., Lima, I. D., Doney, S. C., Mahowald, N., Labiosa, R., and Post, A. F. (2009). Toxicity of atmospheric aerosols on marine phytoplankton. *Proceedings of the National Academy of Sciences*, 106(12):4601–4605.

[Ratnarajah et al., 2014] Ratnarajah, L., Bowie, A. R., Lannuzel, D., Meiners, K. M., and Nicol, S. (2014). The Biogeochemical Role of Baleen Whales and Krill in Southern Ocean Nutrient Cycling. *PLOS ONE*, 9(12):e114067.

[Resing et al., 2015] Resing, J. A., Sedwick, P. N., German, C. R., Jenkins, W. J., Moffett, J. W., Sohst, B. M., and Tagliabue, A. (2015). Basin-scale transport of hydrothermal dissolved metals across the South Pacific Ocean. *Nature*, 523(7559):200–203. Number: 7559 Publisher: Nature Publishing Group.

[Richon et al., 2020] Richon, C., Aumont, O., and Tagliabue, A. (2020). Prey Stoichiometry Drives Iron Recycling by Zooplankton in the Global Ocean. *Frontiers in Marine Science*, 7. Publisher: Frontiers.

[Richon and Tagliabue, 2019] Richon, C. and Tagliabue, A. (2019). Insights Into the Major Processes Driving the Global Distribution of Copper in the Ocean From a Global Model. *Global Biogeochemical Cycles*, 33(12):1594–1610. _eprint: https://agupubs.onlinelibrary.wiley.com/doi/pdf/10.1029/2019GB006280.

[Schlitzer et al., 2018] Schlitzer, R., Anderson, R. F., Dodas, E. M., Lohan, M., Geibert, W., Tagliabue, A., Bowie, A., Jeandel, C., Maldonado, M. T., Landing, W. M., Cockwell, D., Abadie, C., Abouchami, W., Achterberg, E. P., Agather, A., Aguliar-Islas, A., van Aken, H. M., Andersen, M., Archer, C., Auro, M., de Baar, H. J., Baars, O., Baker, A. R., Bakker, K., Basak, C., Baskaran, M., Bates, N. R., Bauch, D., van Beek, P., Behrens, M. K., Black, E., Bluhm, K., Bopp, L., Bouman, H., Bowman, K., Bown, J., Boyd, P., Boye, M., Boyle, E. A., Branellec, P., Bridgestock, L., Brissebrat, G., Browning, T., Bruland, K. W., Brumsack, H.-J., Brzezinski, M., Buck, C. S., Buck, K. N., Buesseler, K., Bull, A., Butler, E., Cai, P., Mor, P. C., Cardinal, D., Carlson, C., Carrasco, G., Casacuberta, N., Casciotti, K. L., Castrillejo, M., Chamizo, E., Chance, R., Charette, M. A., Chaves, J. E., Cheng, H., Chever, F., Christl, M., Church, T. M., Closset, I., Colman, A., Conway, T. M., Cossa, D., Croot, P., Cullen, J. T., Cutter, G. A., Daniels, C., Dehairs, F., Deng, F., Dieu, H. T., Duggan, B., Dulaquais, G., Dumousseaud, C., Echegoyen-Sanz, Y., Edwards, R. L., Ellwood, M., Fahrbach, E., Fitzsimmons, J. N., Russell Flegal, A., Fleisher, M. Q., van de Flierdt, T., Frank, M., Friedrich, J., Fripiat, F., Fröllje, H., Galer, S. J. G., Gamo, T., Ganeshram, R. S., Garcia-Orellana, J., Garcia-Solsona, E., Gault-Ringold, M., George, E., Gerringa, L. J. A., Gilbert, M., Godoy, J. M., Goldstein, S. L., Gonzalez, S. R., Grissom, K., Hammerschmidt, C., Hartman, A., Hassler, C. S., Hathorne, E. C., Hatta, M., Hawco, N., Hayes, C. T., Heimbürger, L.-E., Helgoe, J., Heller, M., Henderson, G. M., Henderson, P. B., van Heuven, S., Ho, P., Horner, T. J., Hsieh, Y.-T., Huang, K.-F., Humphreys, M. P., Isshiki, K., Jacquot, J. E., Janssen, D. J., Jenkins, W. J., John, S., Jones, E. M., Jones, J. L., Kadko, D. C., Kayser, R., Kenna, T. C., Khondoker, R., Kim, T., Kipp, L., Klar, J. K., Klunder, M., Kretschmer, S., Kumamoto, Y., Laan, P., Labatut, M., Lacan, F., Lam, P. J., Lambelet, M., Lamborg, C. H., Le Moigne, F. A. C., Le Roy, E., Lechtenfeld, O. J., Lee, J.-M., Lherminier, P., Little, S., López-Lora, M., Lu, Y., Masque, P., Mawji, E., Mcclain, C. R., Measures, C., Mehic, S., Barraqueta, J.-L. M., van der Merwe, P., Middag, R., Mieruch,

S., Milne, A., Minami, T., Moffett, J. W., Moncoiffe, G., Moore, W. S., Morris, P. J., Morton, P. L., Nakaguchi, Y., Nakayama, N., Niedermiller, J., Nishioka, J., Nishiuchi, A., Noble, A., Obata, H., Ober, S., Ohnemus, D. C., van Ooijen, J., O'Sullivan, J., Owens, S., Pahnke, K., Paul, M., Pavia, F., Pena, L. D., Peters, B., Planchon, F., Planquette, H., Pradoux, C., Puigcorbé, V., Quay, P., Queroue, F., Radic, A., Rauschenberg, S., Rehkämper, M., Rember, R., Remenyi, T., Resing, J. A., Rickli, J., Rigaud, S., Rijkenberg, M. J. A., Rintoul, S., Robinson, L. F., Roca-Martí, M., Rodellas, V., Roeske, T., Rolison, J. M., Rosenberg, M., Roshan, S., Rutgers van der Loeff, M. M., Ryabenko, E., Saito, M. A., Salt, L. A., Sanial, V., Sarthou, G., Schallenberg, C., Schauer, U., Scher, H., Schlosser, C., Schnetger, B., Scott, P., Sedwick, P. N., Semiletov, I., Shelley, R., Sherrell, R. M., Shiller, A. M., Sigman, D. M., Singh, S. K., Slagter, H. A., Slater, E., Smethie, W. M., Snaith, H., Sohrin, Y., Sohst, B., Sonke, J. E., Speich, S., Steinfeldt, R., Stewart, G., Stichel, T., Stirling, C. H., Stutsman, J., Swarr, G. J., Swift, J. H., Thomas, A., Thorne, K., Till, C. P., Till, R., Townsend, A. T., Townsend, E., Tuerena, R., Twining, B. S., Vance, D., Velazquez, S., Venchiarutti, C., Villa-Alfageme, M., Vivancos, S. M., Voelker, A. H. L., Wake, B., Warner, M. J., Watson, R., van Weerlee, E., Alexandra Weigand, M., Weinstein, Y., Weiss, D., Wisotzki, A., Woodward, E. M. S., Wu, J., Wu, Y., Wuttig, K., Wyatt, N., Xiang, Y., Xie, R. C., Xue, Z., Yoshikawa, H., Zhang, J., Zhang, P., Zhao, Y., Zheng, L., Zheng, X.-Y., Zieringer, M., Zimmer, L. A., Ziveri, P., Zunino, P., and Zurbrick, C. (2018). The GEOTRACES Intermediate Data Product 2017. *Chemical Geology*, 493:210–223.

[Semeniuk et al., 2015] Semeniuk, D. M., Bundy, R. M., Payne, C. D., Barbeau, K. A., and Maldonado, M. T. (2015). Acquisition of organically complexed copper by marine phytoplankton and bacteria in the northeast subarctic Pacific Ocean. *Marine Chemistry*, 173:222–233.

[Tagliabue et al., 2018] Tagliabue, A., Hawco, N. J., Bundy, R. M., Landing, W. M., Milne, A., Morton, P. L., and Saito, M. A. (2018). The Role of External Inputs and Internal Cycling in Shaping the Global Ocean Cobalt Distribution: Insights From the First Cobalt Biogeochemical Model. *Global Biogeochemical Cycles*, 32(4):594–616. _eprint: https://agupubs.onlinelibrary.wiley.com/doi/pdf/10.1002/2017GB005830.

[Twining and Baines, 2013] Twining, B. S. and Baines, S. B. (2013). The Trace Metal Composition of Marine Phytoplankton. *Annual Review of Marine Science*, 5(1):191–215.

[van Hulten et al., 2018] van Hulten, M., Dutay, J.-C., and Roy-Barman, M. (2018). A global scavenging and circulation ocean model of thorium-230 and protactinium-231 with improved particle dynamics (NEMO–ProThorP 0.1). *Geoscientific Model Development*, 11(9):3537–3556.

[Weber et al., 2018] Weber, T., John, S., Tagliabue, A., and DeVries, T. (2018). Biological uptake and reversible scavenging of zinc in the global ocean. *Science*, 361(6397):72–76.

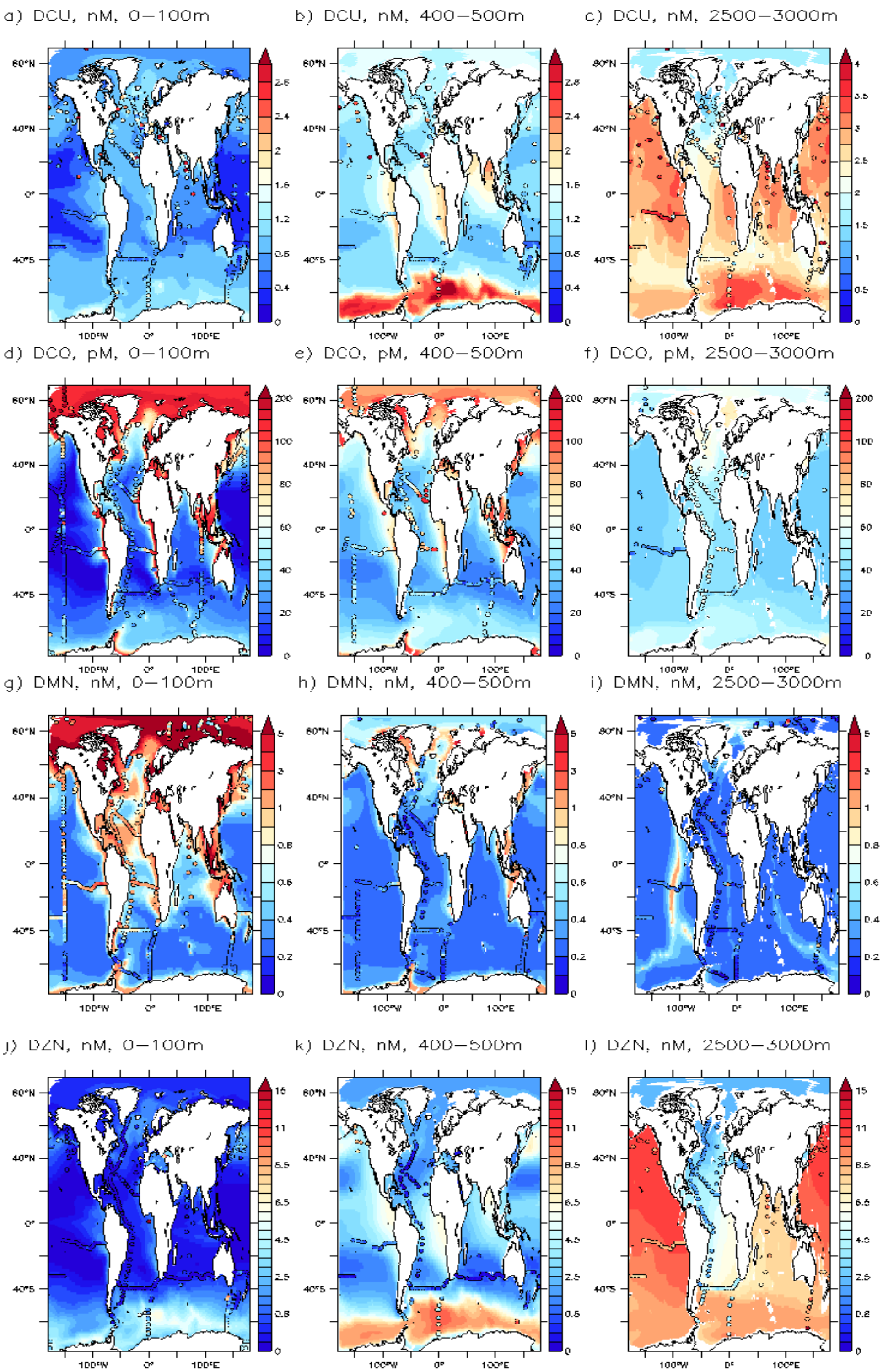

Figure S1: Maps of dissolved micronutrients at different depth. Dots represent data from the GEOTRACES Intermediate Data Product 2017 [Schlitzer et al., 2018] and background colours represent the simulated micronutrient concentrations, as annual averages and averaged over the historical period (1986-2005).

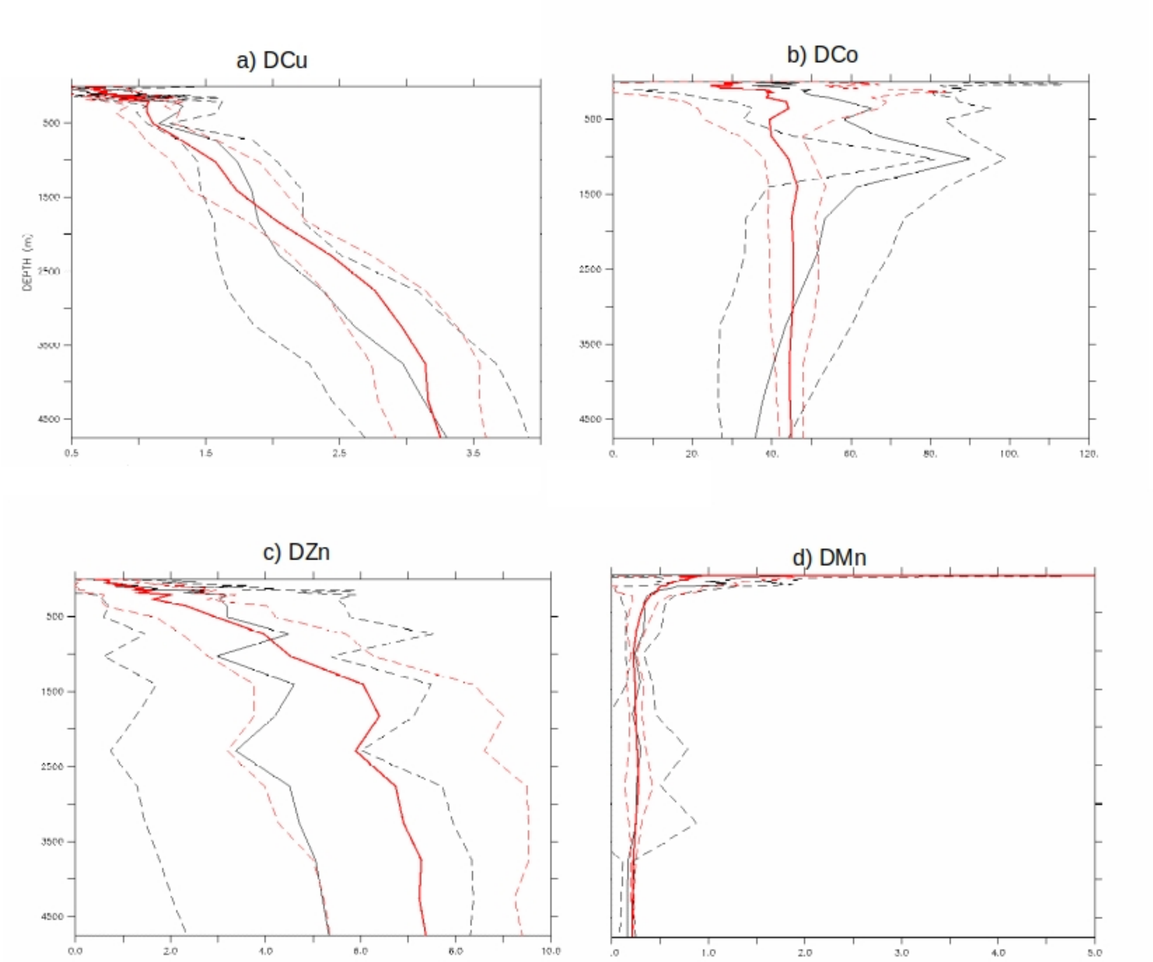

Figure S2: Average micronutrient profiles from the data (black line) and from the model (red line). Dashed lines represent the standard deviation. Model results averaged over the historical period (sensu CMIP: 1986-2005).

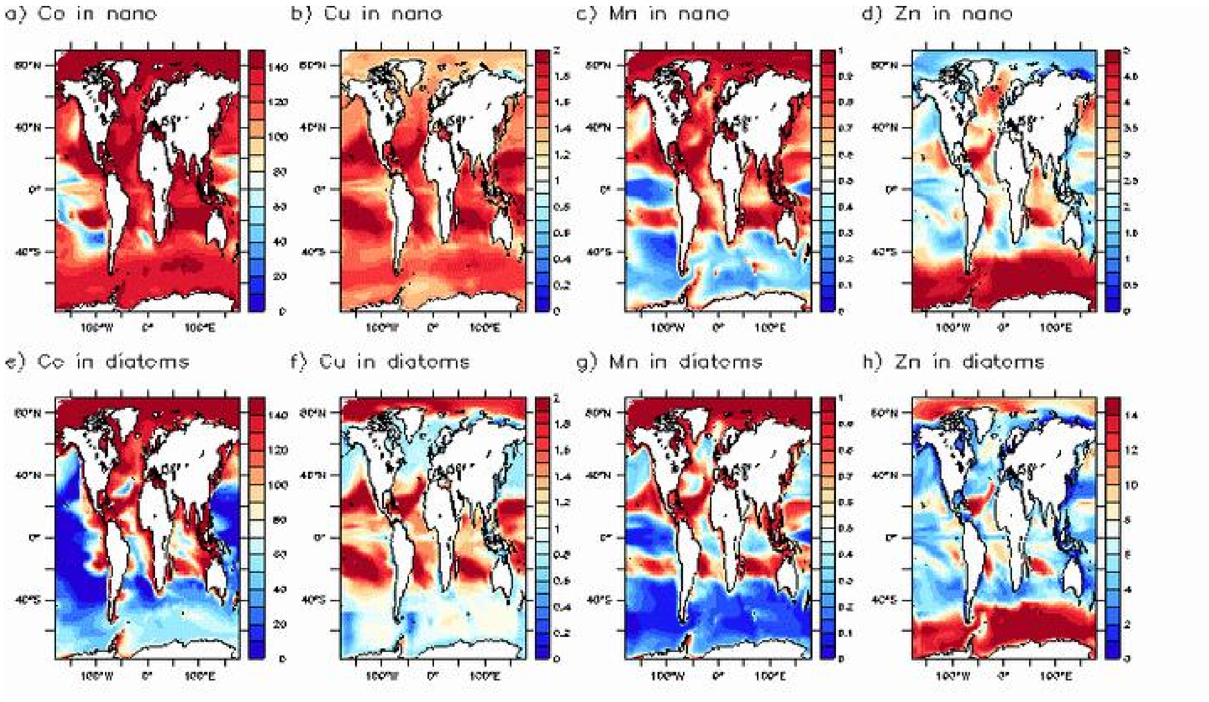

Figure S3: Maps of surface (0-100m) micronutrient:P quotas in nanophytoplankton (a-d) and diatoms (e-h). All values are in mmol/molP except for Co (in μmolCo:molP).

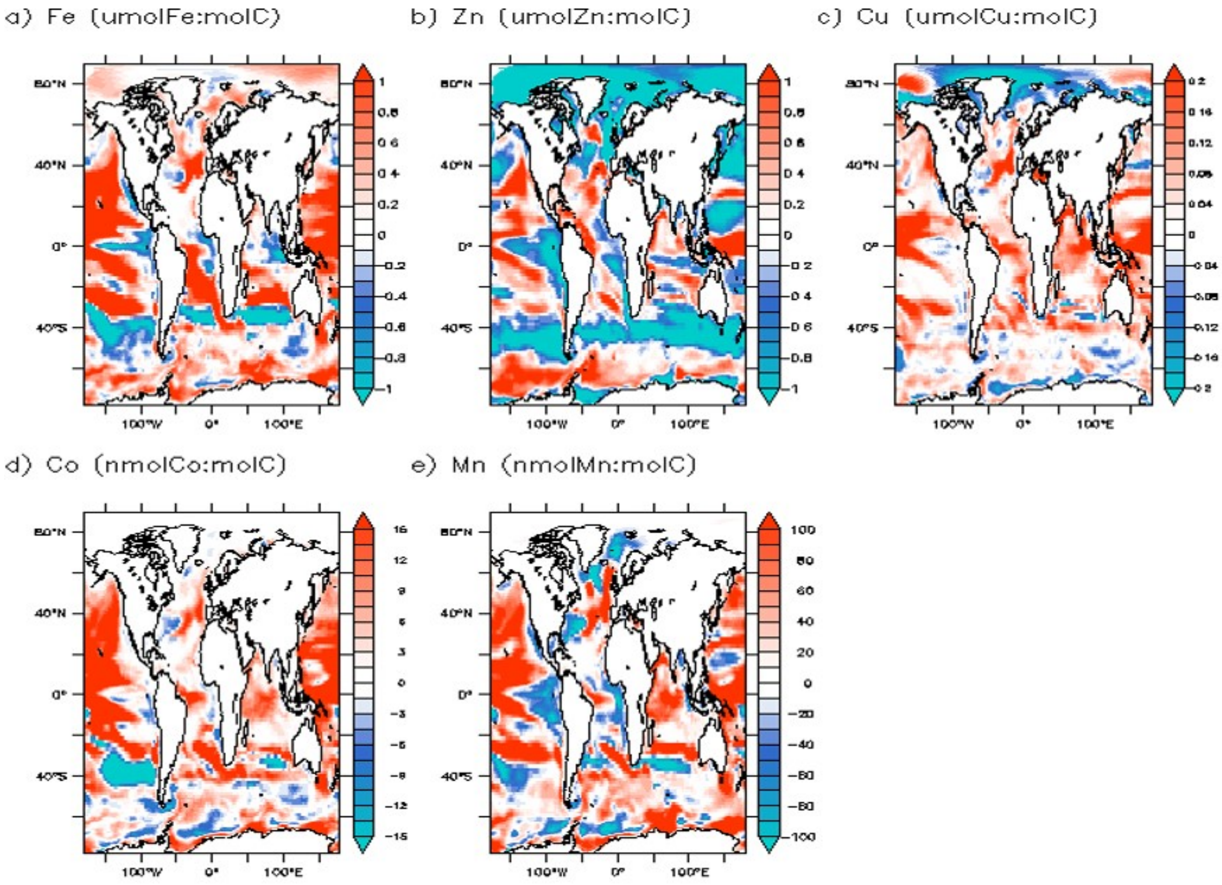

Figure S4: Maps of the changes in surface micronutrient quota in phytoplankton (nanophytoplankton + diatom) cells between the FUTURE and the PRESENT.

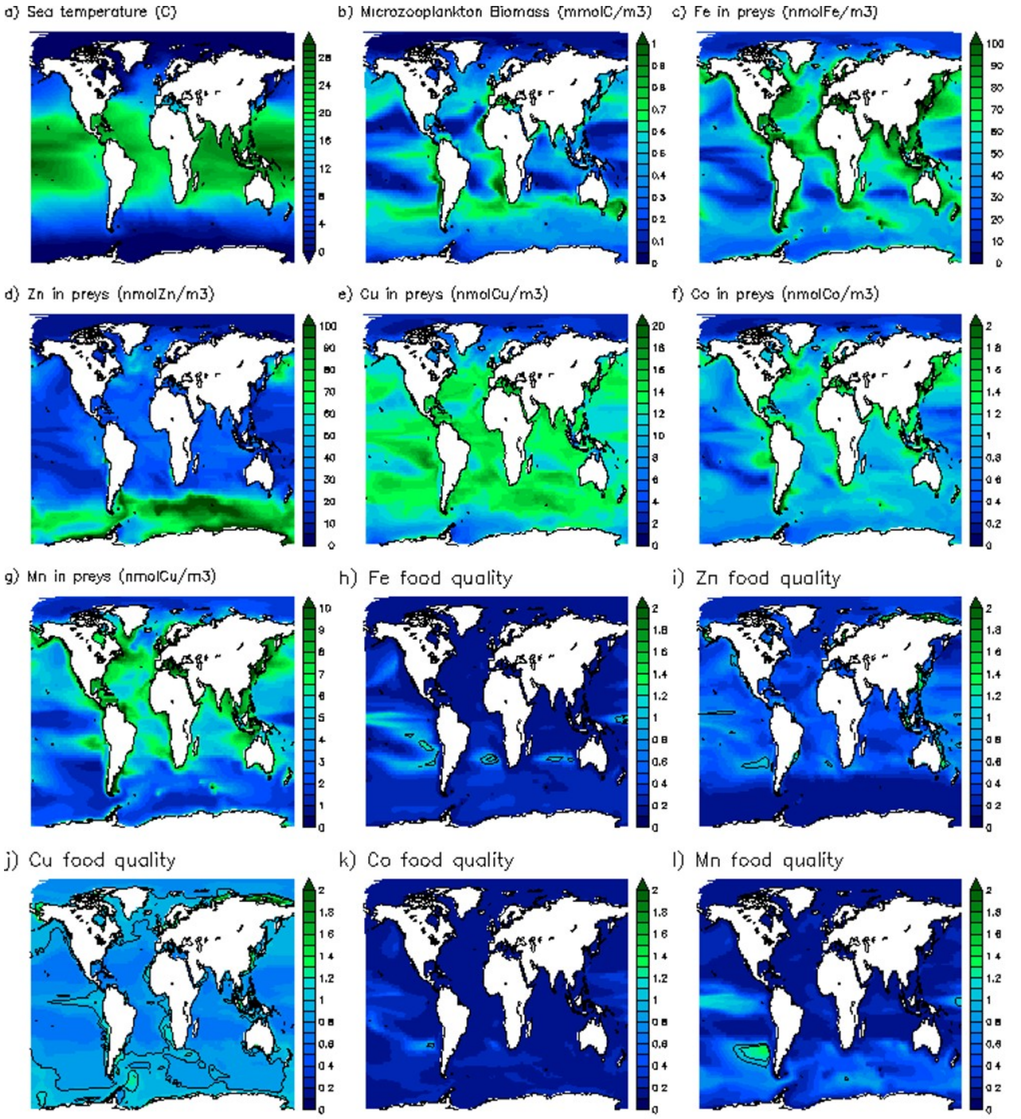

Figure S5: Microzooplankton recycling drivers, averaged in the first 100 meters for the PRESENT (1991-2000) period

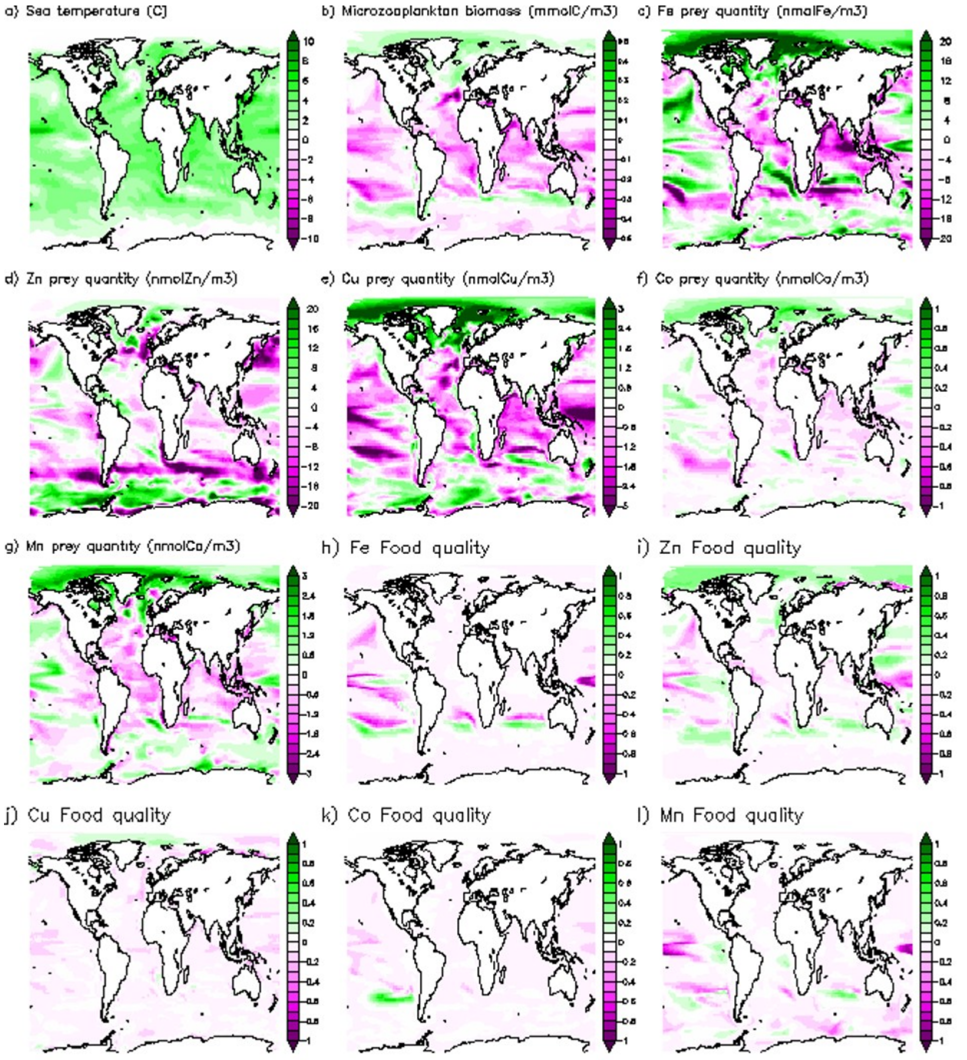

Figure S6: Changes in microzooplankton recycling drivers, averaged in the first 100 meters, between PRESENT and FUTURE. Green colors indicate that the food quality is improving by the end of the century (closer to 1), magenta colors indicate food quality is decreasing (a-e).
